# Imatinib alternating with regorafenib compared to imatinib alone for the first-line treatment of advanced gastrointestinal stromal tumor: The AGITG ALT-GIST intergroup randomized phase II trial

**DOI:** 10.1038/s41416-025-02983-w

**Published:** 2025-03-25

**Authors:** Desmond Yip, John Zalcberg, Jean-Yves Blay, Mikael Eriksson, David Espinoza, Timothy Price, Sandrine Marreaud, Antoine Italiano, Neeltje Steeghs, Kjetil Boye, Craig Underhill, Val Gebski, John Simes, Hans Gelderblom, Heikki Joensuu

**Affiliations:** 1 The Canberra Hospital and Australian National University, Canberra, ACT Australia; 2 School of Public Health, Monash University, Melbourne, VIC Australia; 3 Centre Léon Bérard, Lyon, France; 4 Skane University Hospital and Lund University, Lund, Sweden; 5 NHMRC Clinical Trials Centre, The University of Sydney, Sydney, NSW Australia; 6 Queen Elizabeth Hospital, University of Adelaide, Adelaide, SA Australia; 7 European Organisation for Research and Treatment of Cancer (EORTC), Brussels, Belgium; 8 Institut Bergonié, Bordeaux, France; 9 Department of Medical Oncology, The Netherlands Cancer Institute, Antoni van Leeuwenhoek Hospital, Amsterdam, The Netherlands; 10 Oslo University Hospital, Oslo, Norway; 11 Albury-Wodonga Regional Cancer Centre, Albury-Wodonga, NSW Australia; 12 Leiden University Medical Centre, Department of Medical Oncology, Leiden, The Netherlands; 13 Helsinki University Hospital Comprehensive Cancer Center, Helsinki, Finland

**Keywords:** Sarcoma, Drug development

## Abstract

**Background:**

To determine if an alternating regimen of the tyrosine kinase inhibitors imatinib and regorafenib improved outcomes in patients with advanced gastrointestinal stromal tumors.

**Methods:**

ALTGIST (NCT02365441) was a randomized phase II study of standard treatment of imatinib (Arm A) compared with an experimental alternating regimen of imatinib and regorafenib (Arm B). Primary outcome was best objective tumor response (OTR) at nine months.

**Results:**

Seventy-six eligible patients (Arm A 36, Arm B 40) enrolled were evaluable. Median follow-up was 46.0 months (range 6.5–64.6). Best responses and OTR were similar at 9 months. Eighteen (50.0%) Arm A patients and twelve (30.0%) Arm B patients discontinued treatment due to progressive disease. No Arm A patients stopped protocol therapy due to unacceptable toxicity, with 12 (30.0%) stopping in Arm B. Twelve (33.2%) Arm A patients and 12 (30.0%) Arm B patients experienced at least one serious adverse event, mostly grade 3. Secondary endpoints of PFS at 1 and OS at 1 year were not statistically different.

**Conclusions:**

Alternation of imatinib and regorafenib did not impact on 9 months objective response nor on the secondary objectives of PFS and OS. Patients in the alternating arm experienced more toxicity and protocol discontinuations.

**Clinical trial registration:**

NCT02365441.

## Background

While modern treatment of metastatic gastrointestinal stromal tumors (GIST) with imatinib (Glivec, Gleevec) is highly active, with clinical benefit rates of over 80%, most patients will ultimately progress. Two large pivotal randomized trials of 400 mg versus 800 mg of imatinib conducted in Europe/Australasia and North America respectively [1, 2], demonstrated that the median progression-free survival (PFS) was approximately two years. However, more recent data suggests that the median PFS is longer than this in patient populations with a smaller tumor burden at the time of imatinib initiation [3]. The overall median survival of patients is currently at least five years [4]. The risk of progression and death can be estimated by a prognostic nomogram that takes into account factors such as tumor genotype, primary mitotic count, size of metastasis, hemoglobin and neutrophil count at start of treatment [5].

Tumors may express varying mechanisms of resistance to imatinib [6] as well as second-generation tyrosine kinase inhibitors (TKI), due either to the acquisition of secondary mutations in *KIT* [7] or potentially the selection of pre-existing drug-resistant clones [8]. The emergence of resistance to imatinib occurs despite initial response rates of well over 80% in the more sensitive tumors carrying mutations in exon 11 of *KIT*. Imatinib treatment interruption and resumption at progression was not found to impact on resistance to imatinib in a randomized trial testing interruption at 1, 3, and 5 years [9].

Given the exquisite sensitivity of GIST cells to imatinib in vitro [10], and the dramatic clinical effect of imatinib administered to people in the first-line setting [11], particularly in tumors carrying mutations in *KIT* exon 11 (representing approximately 65% of all patients), it is unclear why complete responses are comparatively rare and the cure rate so low, with the vast majority of patients ultimately dying with advanced disease. Primary resistance to imatinib which is relatively uncommon may be due to the patients having *KIT* exon 9, *PDGFR* D842V mutations or no *KIT/PDGFR* mutations. The majority of GIST tumors progressing on imatinib therapy demonstrate the development of secondary *KIT* or *PDGFR* mutations which are resistant to the tyrosine kinase inhibitor. These commonly involve either the kinase activation loop (exons 17 and 18) or the ATP-binding pocket (exons 13 and 14) conferring imatinib resistance [12].

We hypothesized that by eliminating the selective pressure of continued pharmacologic therapy — which encourages the proliferation of resistant clones — by temporarily discontinuing treatment (a “washout” period), and using a second TKI such as regorafenib with established activity in GIST related in part to additional mechanisms of action beyond inhibition of KIT,^13,^ response rates and survival times may be improved. Tumor cells harboring secondary mutations that expand predominantly because of this selective pressure might also regress during a brief period free of TKI exposure and become more susceptible to therapy.

Given the above rationale, it was hypothesized that with the addition of regorafenib (Stivarga), a second targeted agent with very substantial activity in the third-line setting in GIST [13], residual tumor cells may be eradicated and some partial responders might be converted into complete responders after a brief drug-free period. Regorafenib [14] was chosen as it represents a TKI with inhibitory activity against VEGFR1-3, FGFR, PDGFRA, Tie 2, BRAF, RET, RAF and p38 MAPK signaling pathways in addition to KIT. Secondary *KIT* mutations of exon 17 [15] and 18 [16] may also be sensitive to regorafenib. In a randomized, placebo controlled phase III trial in 199 patients who had failed at least two previous lines of therapy for GIST, there was a statistically significant reduction in the risk of progression in the regorafenib arm compared to the placebo arm (median PFS 4.8 vs. 0.9 months, HR 0.27) [17].

These strategies for improving response to therapy (treatment-free periods and addition of a second active agent) were incorporated in the design of this pragmatic trial, which was originally aimed to increase the OTR and PFS rates and ultimately increase the overall survival (OS) rate for advanced GIST.

## Methods

### Study design and participants

This phase II, randomized 1:1, open-label trial was conducted across 37 centers in 11 countries (Australia, Finland, France, Italy, Netherlands, Norway, Singapore, Slovakia, Spain, Sweden, United Kingdom). The trial sponsor was the Australasian Gastro-Intestinal Trials Group (AGITG) with collaborating groups being the AGITG, the European Organisation for Research and Treatment of Cancer (EORTC) and the Scandinavian Sarcoma Group (SSG). The study was coordinated by the National Health and Medical Research Council (NHMRC) Clinical Trials Centre, University of Sydney, Australia. See Supplemental Appendix for details of participating sites, investigators and contributing personnel. Patients aged 18 years or older, with a confirmed diagnosis of CD-117 positive, unresectable, metastatic GIST were eligible for this trial. Patients with CD-117 negative disease had to be DOG-1 positive or *KIT/PDGFRA* mutated. Patients with Eastern Cooperative Oncology Group performance status of 0 to 2 and adequate organ function were included. Patients were required to have measurable disease by Response Evaluation Criteria in Solid Tumors (RECIST) version 1.1. Patients must have had no prior history of taking a TKI for metastatic disease, with the exception of those patients who had up to and including 21 days of uninterrupted treatment on 400 mg daily of imatinib; imatinib therapy given as an adjuvant treatment and completed at least three months prior to entry into this trial was permitted. Patients who had progression of GIST while on adjuvant therapy were not eligible for this trial.

Key exclusion criteria included poorly controlled hypertension or persistent proteinuria of >3.5 g/24 h; inability to swallow or malabsorption; an arterial or venous thrombotic event within six months prior to randomization; known central nervous system metastases; active hepatitis B or C or HIV infection, and presence of known *PDGFRA* D842V mutation or other mutation known to cause imatinib resistance.

The trial was registered at ClinicalTrials.gov as NCT02365441.

### Randomization

This was an open-label trial with central randomization by the NHMRC Clinical Trials Centre via a web-based system 1:1 by minimization to continuous imatinib (Arm A) or to alternating imatinib with regorafenib (Arm B). The randomization was stratified by site, receipt of previous adjuvant therapy (prior vs. none), and receipt of imatinib for metastatic disease for less than or equal to 21 days.

### Procedures

Imatinib was administered orally at 400 mg once daily continuously on a 56-day cycle. In Arm B imatinib was administered at 400 mg orally daily for 21–25 days followed by a washout (drug-free) period of 3–7 days, then regorafenib 160 mg once daily orally for three weeks followed by a seven-day washout period. Treatment was continued until disease progression as per RECIST 1.1 or unacceptable adverse events.

Study treatment was delayed or stopped in case of occurrence of adverse events and resumed once protocol-defined criteria were met. See study protocols in Supplementary Appendix documents. Dose delays and reductions were permitted for imatinib and regorafenib for toxicities prespecified in the protocol.

Tumor assessment was by CT scan at baseline and every eight weeks for the first year on trial, and then every 12 weeks until disease progression or death, timed from the randomization date. PET scans were performed for Arm B patients enrolled into the PET substudy, patients were randomized to receive two PET scans either during the imatinib or regorafenib washout period one week apart, results yet to be reported. Hematology, biochemistry, and urine dipstick were performed before Day 1 of each cycle. Adverse events were recorded at each visit according to National Cancer Institute Common Terminology Criteria for Adverse Events (CTCAE) version 4.03.

Blood for pharmacokinetic analysis were taken as follows: for imatinib levels, plasma samples were taken on Week 4, Day 1 of Cycles 1 and 2 in Arm A, and Week 3 (last day of imatinib administration) of Cycles 1 and 2 in Arm B; for regorafenib levels, plasma samples were taken on Week 7 (last day of regorafenib administration) of Cycles 1 and 2 in Arm B only. Serial blood samples were also collected at other timepoints for exploratory biomarker studies.

### Outcomes

The primary outcome, objective tumor response (OTR) at 9 months, was measured, as defined by RECIST 1.1 (complete or partial response) and assessed by the investigator with central confirmation of response at or before nine months from the time of either (i) randomization or (ii) commencement of therapy (if patients were randomized during their first cycle of imatinib). Secondary outcomes were OS, PFS, treatment-related safety, toxicity, tolerability, clinical benefit rate (stable disease + partial response + complete response) following three cycles (24 weeks) of treatment, and time to treatment failure. OS was defined as the interval from either (i) randomization (if patients had not yet commenced treatment) or (ii) commencement of therapy (if patients were randomized during the first cycle of imatinib) to date of death from any cause, or the date of last known follow-up alive. PFS was the length of time from either (i) randomization (if patients had not yet commenced treatment) or (ii) commencement of therapy (if patients were randomized during the first cycle of imatinib) until disease progression defined according to RECIST 1.1 or death. Patients were censored at last known status if progressive disease (PD) had not yet occurred or censored if switched to non-protocol therapy; patients were still continued to be followed to PD if still on any part of protocol therapy (eg: imatinib alone); surgery for residual disease prior to PD was allowed as part of protocol therapy (so patients were still followed further for PD).

Patients who had received at least one dose of the study drugs were included in evaluation of treatment safety.

The clinical benefit rate was calculated by summing the number of participants assessed as having a complete response, partial response, or stable disease within the first 24 weeks from randomization, and dividing this by the total number of participants evaluable for response (according to RECIST version 1.1) within the first 24 weeks. Time to treatment failure was defined as the time from either (i) randomization (if patients had not yet commenced treatment) or (ii) commencement of therapy (if patients were randomized during their first cycle of imatinib) to treatment discontinuation for any reason, including disease progression, treatment toxicity, patient preference, or death.

### Statistical analysis

The study was originally planned to enroll 240 patients in a 1:1 randomization ratio. Analyses were to include all patients who were randomized. Treatment activity was to be assessed by the proportion, together with the 95% confidence interval, of patients not progressing at 24 months. In order to demonstrate a relative increase in progression free survival (PFS) at 24 months from the date of randomization from an expected 78% to 88%, with 80% power and 95% confidence based on A’Hern’s adjustment to Fleming’s design, approximately 110 evaluable participants were required in each arm. Thus 240 participants were proposed allowing for approximately a 10% drop-out rate. Eighty percent of participants were expected to achieve a clinical benefit at 24 months (CBR – rate of complete or partial response, or stable disease). A secondary outcome was to determine whether a minimum 25% relative increase of the CBR in the experimental cohort could be attained.

Due to logistical difficulties with opening centers to recruitment across multiple countries in Europe and Asia as well as slow enrollment, the funding available for this study was reduced, necessitating a revision of the recruitment target to 76 patients in a 1:1 randomization ratio in July 2017. The primary endpoint was altered to the OTR at or before nine months as it was uncertain at that date what amount of funding would be available to continue followup of patients enrolled and data collection. Time to treatment failure was altered to consider events in the first 12 months. Patients not experiencing treatment failure within 12 months would be censored at 12 months. PFS and overall survival at one year were new secondary objectives. These changes were made with the process being blinded to trial outcome data by randomized group. Based on a 95% one-sided confidence interval, for different levels of the true underlying response rate, the minimum number of responses required to be observed in the alternating group to classify the alternating regimen as active was as follows: 40%, ≥ 10 responses; 50%, ≥ 13 responses; 60%, ≥ 17 responses; 70%, ≥ 22 responses.

### Role of funding source

The funder of the study had no role in the study design, study execution, data collection, data analyses, data interpretation or decision to submit results for publication.

## Results

Between June 2015 and September 2018, 78 patients were screened, of whom two were excluded from randomization. We randomly assigned 76 patients, with 36 in the Arm A (imatinib only) and 40 in Arm B (imatinib alternating with regorafenib) (Table 1). Baseline disease characteristics and demographic characteristics are given in Table 1. The patients were predominately male in both arms. The mean age was 63.1 in Arm A and 59.3 in Arm B, with the stomach the most common primary site in both Arm A (38.9%) and Arm B (35.0%). Exon 11 and 9 *KIT* mutations were the most common alterations.

**Table 1:** Baseline demographics.

	Imatinib only *n* = 36 (%)	Alternating therapy *n* = 40 (%)
Gender		
Female	16 (44)	9 (23)
Male	20 (56)	31 (78)
Mean age (SD), years	63.1 (11.2)	59.3 (12.1)
Primary tumor site		
Stomach	14 (39)	14 (35)
Small intestine	9 (25)	16 (40)
Colon/Rectum	3 (8)	1 (3)
Peritoneum/Omentum/Mesentery	2 (6)	2 (5)
Pelvis	1 (3)	1 (3)
Not possible to define	7 (19)	6 (15)
Metastasis at initial diagnosis		
Yes	22 (61)	24 (60)
No	14 (39)	16 (40)
Liver metastases		
Yes	25 (69)	22 (55)
No	11 (31)	18 (45)
Intra-abdominal metastases		
Yes	18 (50)	16 (40)
No/NA	18 (50)	24 (60)
Bone metastases		
Yes	1 (3)	1 (3)
No/NA	35 (97)	39 (97)
Lung metastases		
Yes	1 (3)	2 (5)
No	35 (97)	38 (95)
Mutation result		
*KIT* exon 11*	26 (72)	33 (83)
*KIT* exon 9	3 (8)	3 (8)
KIT exon 13 and 17*	1 (3)	0 (0)
*PDGFRA* exon 12	0 (0)	1 (3)
*PDGFRA* exon 18	0 (0)	1 (3)
*KIT/PDGFRA* negative	3 (8)	2 (5)
No mutation results available	3 (8)	0 (0)
Tumor rupture		
Yes	1 (3)	2 (5)
No	35 (97)	38 (95)
Prior adjuvant Imatinib therapy		
Yes	10 (28)	12 (30)
No	26 (72)	28 (70)
Commenced Imatinib for metastatic disease prior to Randomization (as per protocol)?		
Yes	13 (36)	15 (38)
No	23 (64)	25 (63)

^*^Two patients had dual primary mutations: exon 11 and 4 and exon 13 and 17 (both Arm A).

At data cut off, on 31 December 2020, median follow-up time was 46.0 months (range 6.5– 64.6 months) for all patients. Relative dose intensity was 95.0% for imatinib in Arm A and 98.9% for imatinib and 84.1% for regorafenib in Arm B. The best responses to Arm A and Arm B treatments were similar at nine months; 0 vs. 1 patients had complete response, 22 vs. 23 partial response, 14 vs. 15 stable disease (Table 2). The primary endpoint of OTR at or before nine months was 61.1% (95% CI, 44.9–75.2%) and 60.0% (95% CI, 44.6–73.7%), respectively, in Arm A and Arm B. Estimated clinical benefit rate (CR + PR + SD) at three cycles (24 weeks) was achieved in 97.2% patients in Arm A (95% CI 85.8–99.5%) and 87.5% in Arm B (95% CI 73.9–94.5%).

**Table 2:** Estimated objective tumor response rate at nine months.

Response	Imatinib only *n* = 36 (%)	Alternating therapy *n* = 40 (%)
Complete response	0 (0)	1 (3)
Partial response	22 (61)	23 (58)
Stable disease	14 (39)	15 (38)
Progressive disease	0 (0)	1 (3)

There were 8 (22.2%; 95% CI, 11.7–38.1%) and 6 (15.0%; 95% CI, 7.1–29.1%) patients respectively in Arm A and Arm B who underwent surgical resection of residual disease. Of these patients 7 had complete excision of disease and 6 incomplete excision. Two patients had disease progression prior to surgery and one had progression after surgery. Eight patients received non protocol systemic therapy after surgery.

**Fig. 1:**
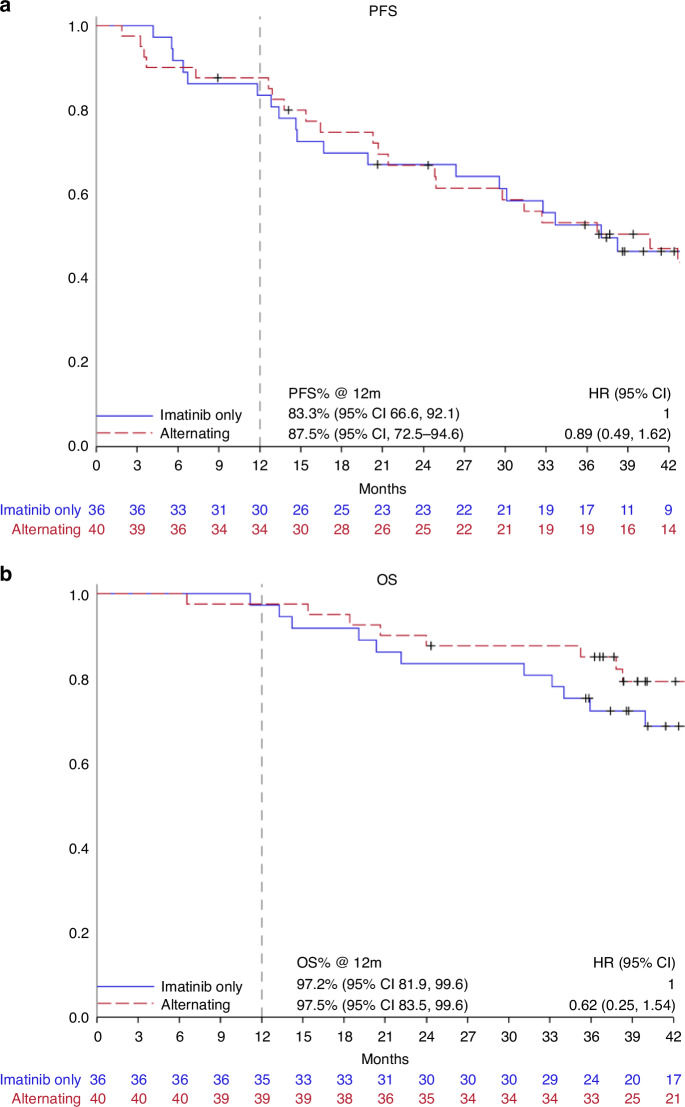
Patient survivals of Arm A versus Arm B. Kaplan Meier survival curves for progression free Panel **a** and overall survival Panel **b**.

At data cut off, on 31 December 2020, 12 (33.2%) patients in Arm A and 9 (22.5%) patients in Arm B were still on study treatment; 18 patients (50.0%) in Arm A and 12 patients (30.0%) in Arm B had discontinued protocol therapy due to progressive disease. The secondary endpoint of PFS at one year was Arm A 83.3% (95% CI 66.6–92.1) and Arm B 87.5% (95% CI, 72.5–94.6), p_logrank_ = 0.71. The median PFS was 37.0 months and 36.7 months in arm A and arm B, respectively. OS at one year was 97.2% (95% CI 81.9–99.6) and 97.5% (95% CI 84.5–99.6) respectively; p_log rank_ = 0.30. The median overall survival was not reached in any arm. The Kaplan-Meier curves for PFS and OS are shown in Fig. 1.

An exploratory subgroup analysis was performed with respect to CBR, PFS and OS according to the exon 11 *KIT* mutation status. In patients with exon 11 *KIT* mutation (n = 60, 27 Arm A and 33 Arm B) the CBR were 100% (95% CI 87.5–100) and 93.9% (95% CI 80.4-98.3) in Arm A (27/27 patients) and Arm B (31/33 patients), respectively (*p* = 0.6); in patient with non-exon 11 *KIT* mutation (*n* = 8), the CBR were 66.7% (95% CI 20.8–93.9) and 80.0% (95% CI 37.6–96.4) in Arms A (2/3 patients) and B (4/5 patients), respectively (*p* = 1.0). In patients with exon 11 *KIT* mutation, the estimated 12-month PFS was 85.2% (95% CI 65.2–94.2) in Arm A and 93.9% (95% CI 77.9–98.4) in Arm B, p_logrank_ = 0.88. In patients with non-exon 11 *KIT* mutation, the estimated 12-months PFS was 33.3% (95% CI 0.9–77.4) in Arm A and 80.0% (95% CI 20.4–96.9) in Arm B, p_logrank_ = 0.26. The OS at one year for patients with exon 11 *KIT* mutation was 96.3% (95% CI 76.5–99.5) in Arm A and 100% (95% CI not estimable) in Arm B, p_logrank_ = 0.79. The OS at one year for patients with non-exon 11 *KIT* mutation was 100% (95% CI not estimable) in both Arm A and Arm B, p_logrank_ = 0.14. The mutation analysis results of the cohort are shown in Table 1.

Kaplan-Meier estimate of treatment failure at 12 months was 83.3% (66.7–92.1) for Arm A and 75.0% (58.5–85.7) for Arm B, p_logrank_ = 0.36 with a hazard ratio of 1.60 (0.58–4.39), alternating versus imatinib only.

As data collection was able to be continued beyond the data cutoff of December 2020, an updated analysis was carried out of PFS and OS as of the last available date of May 18, 2023. This found no difference in PFS or OS. At four years the PFS rates were Arm A: 40.0% (23.8-55.7) and Arm B 35.8% (20.8–0.51.0) p_logrank_ = 0.68 and estimated OS rates were: 69.1% (51.2–81.6) and 74.1% (57.2–85.2) respectively, p_logrank_ = 0.46. See Supplementary Fig. 1 for graph of PFS. In Arm A 14 of the 38 (37%) disease progression (PD) events were due to new lesion development compared to 12 out of 40 (30%) in Arm B.

Duration of response (defined as time from registration to disease progression or death in patients who achieve complete or partial response) was also explored up to the last available follow-up. This found a median duration of response of 57.5 months with Arm A (48.6– 66.8) and 40.2 months with arm B (38.8–49.9), *p* = 0.612. See Supplemental Fig. 2 for graph of PFS in responders.

Treatment-related adverse events of any grade occurred in 36 (100%) of 36 patients in Arm A and 40 (100%) of 40 patients in Arm B. Grade 3 or 4 adverse events occurred in 16 (44.4%) and 23 (57.5%) patients in Arm A and Arm B, respectively (Table 3). The most common grade 3 or 4 adverse events were anemia and diarrhea in Arm A. In Arm B, the most common grade 3 or 4 adverse events were palmar-plantar erythrodysesthesia syndrome and hypertension. Dose reductions occurred in 11 (30.5%) patients in Arm A and 25 (62.5%) patients in Arm B. Hematological toxicity led to dose delay in 2 patients in Arm A and one patient in Arm B due to regorafenib. Twelve patients (30.0%) in Arm B stopped protocol therapy due to unacceptable toxicity, and none in Arm A. Twelve (31.5%) patients in Arm A and 12 (30.0%) in Arm B experienced at least one serious adverse event. The most common serious treatment-related adverse event was diarrhea (3 [7.8%] of 38) in Arm A and abdominal pain (2 [5.0%] of 40) in Arm B.

**Table 3:** Treatment-emergent adverse events of all grades in ≥ 10% of the safety population.

Any adverse event		Imatinib only (*n* = 38)				Alternating (*n* = 40)				
		Grade 1 + 2	Grade 3	Grade 4	Total	Grade 1 + 2	Grade 3	Grade 4	Grade 5	Total
		22 (58%)	13 (34%)	3 (8%)	38 (100%)	17 (43%)	19 (48%)	3 (8%)	1 (3%)	40 (100%)
General	Fever	2 (5%)	0	0	2 (5%)	6 (15%)	0	0	0	6 (15%)
	Anorexia	6 (16%)	0	0	6 (16%)	10 (25%)	0	0	0	10 (25%)
	Fatigue	23 (61%)	0	0	23 (61%)	24 (60%)	5 (13%)	0	0	29 (73%)
	Edema face/periorbital /trunk/limbs	24 (63%)	0	0	24 (63%)	19 (48%)	0	0	0	19 (48%)
	Arthralgia	7 (18%)	0	0	7 (18%)	4 (10%)	0	0	0	4 (10%)
	Myalgia	3 (8%)	0	0	3 (8%)	4 (10%)	0	0	0	4 (10%)
	Back pain	4 (11%)	0	0	4 (11%)	5 (13%)	0	0	0	5 (13%)
	Dizziness	5 (13%)	0	0	5 (13%)	5 (13%)	0	0	0	5 (13%)
	Headache	5 (13%)	0	0	5 (13%)	11 (28%)	0	0	0	11 (28%)
	Insomnia	4 (11%)	0	0	4 (11%)	3 (8%)	0	0	0	3 (8%)
	Vertigo	5 (13%)	0	0	5 (13%)	0 (0%)	0	0	0	0 (0%)
Gastrointestinal disorders	Abdominal distension	5 (13%)	0	0	5 (13%)	1 (3%)	0	0	0	1 (3%)
	Abdominal pain	14 (37%)	1 (3%)	0	15 (39%)	14 (35%)	2 (5%)	0	0	16 (40%)
	Constipation	6 (16%)	0	0	6 (16%)	8 (20%)	0	0	0	8 (20%)
	Diarrhea	19 (50%)	3 (8%)	0	22 (58%)	14 (35%)	4 (10%)	0	0	18 (45%)
	Dry mouth	2 (5%)	0	0	2 (5%)	8 (20%)	0	0	0	8 (20%)
	Nausea	20 (53%)	0	0	20 (53%)	16 (40%)	0	0	0	16 (40%)
	Vomiting	9 (24%)	0	0	9 (24%)	5 (13%)	0	0	0	5 (13%)
Investigations	Increased ALT	2 (5%)	2 (6%)	0	4 (11%)	5 (13%)	0	0	0	5 (13%)
	Increased AST	6 (16%)	0	0	6 (16%)	4 (10%)	0	0	0	4 (10%)
	Increased AP	2 (5%)	0	0	2 (5%)	4 (10%)	0	0	0	4 (10%)
	Increased creatinine	6 (16%)	0	0	6 (16%)	1 (3%)	0	0	0	1 (3%)
	Increased lipase	0 (0%)	1 (3%)	1 (3%)	2 (5%)	0 (0%)	1 (3%)	3 (8%)	0	4 (10%)
	Hypocalcemia	5 (13%)	0	0	5 (13%)	6 (15%)	0	0	0	6 (15%)
	Hypomagnesemia	4 (11%)	0	0	4 (11%)	4 (10%)	0	0	0	4 (10%)
	Hypophosphatemia	5 (13%)	1 (3%)	0	6 (16%)	6 (15%)	4 (10%)	0	0	10 (25%)
	Anemia	10 (26%)	3 (8%)	0	13 (34%)	12 (30%)	0	0	0	12 (30%)
Respiratory	Cough	1 (3%)	0	0	1 (3%)	5 (13%)	0	0	0	5 (13%)
	Upper resp. tract infection	3 (8%)	0	0	3 (8%)	3 (8%)	1 (3%)	0	0	4 (10%)
	Dyspnea	0 (0%)	0	0	0 (0%)	6 (15%)	0	0	0	6 (15%)
Skin and subcutaneous disorders	Alopecia	3 (8%)	0	0	3 (8%)	7 (18%)	0	0	0	7 (18%)
	Dry skin	4 (11%)	0	0	4 (11%)	10 (25%)	0	0	0	10 (25%)
	Palmar-plantar erythrodysesthesia syndrome	1 (3%)	0	0	1 (3%)	25 (63%)	6 (15%)	0	0	31 (78%)
	Maculo-papular rash	5 (13%)	1 (3%)	0	6 (16%)	6 (15%)	2 (5%)	0	0	8 (20%)
Cardiovascular	Hypertension	6 (16%)	0	0	6 (16%)	13 (33%)	8 (20%)	0	0	21 (53%)

Death while on treatment or follow-up occurred in 11 (30.6%) of 36 patients in Arm A and 12 (30.0%) of 40 patients in Arm B. Among these, 9/11 (81.8%) and 9/12 (75.0%) were related to disease progression in Arm A and Arm B, respectively. Death due to study drug toxicity was reported in one patient in Arm B (myocardial infarction) and no patients in Arm A.

## Discussion

While our working hypothesis was that rotation of treatment would limit the emergence of clonal resistance, we observed no difference in the primary endpoint of objective tumor response at 9 months nor on secondary endpoints of PFS and OS in this prospective randomized study testing for the first time the rotation of TKIs as a strategy for first line treatment.

The endpoints were non-significantly different between the alternating therapy arm compared to the imatinib-only arm. In addition, there was no difference in subgroup analysis based on the presence or absence of an exon 11 *KIT* mutation, history of prior imatinib treatment (as adjuvant therapy or before randomization) and the tumor site. Although the observed DFS in Arm B was 80% while in Arm A it was 33.3% in the non-exon 11 patients, this non-significant trend was not accounted for by differences in the numbers of exon 9 and KIT/PDGFRA negative (wild type) patients between the two arms. These two genotypes are sensitive to regorafenib but are relatively less sensitive and resistant to imatinib respectively.

Mutations in the KIT and PDGFRA receptors are critical gain of function drivers in 90% of patients with GIST [18, 19]. Inhibition of these receptors with imatinib results in improved survival in the majority of patients with GIST; however after an initial clinical response, tumor progression occurs in most patients [2]. In around 90% of patients imatinib failure is due to secondary mutations in *KIT* in two regions of the kinase domain: the ATP binding pocket and the activation loop [8, 20]. A preclinical study in imatinib-resistant GIST using alternating regorafenib and sunitinib reported that regorafenib mainly inhibits KIT kinases with a mutation in the activation loop and sunitinib inhibits kinases with an ATP binding pocket mutation [21]. This study suggested that a combination of TKIs may prevent polyclonal imatinib resistance due to the development of secondary mutations. Serano et al. conducted a phase Ib study to investigate the feasibility and tolerability of continuous treatment with rapid alternation of sunitinib for 3 days followed by regorafenib for 4 days in patients with TKI-resistant GIST [22]. They reported stable disease in 31% patients (4/13 patients), a median PFS of 1.9 months (95% CI 1.4–3.6 months) and a median OS of 10.8 months (95% CI, 5.9–∞), and mainly grade 1–2 adverse events. Although this schedule was tolerable and feasible, low plasma drug concentrations of both sunitinib and regorafenib were found below threshold target inhibition concentrations suggesting that clinically meaningful drug exposure was not achieved. As sustained stable disease was seen in the study with clinical benefit it was thought by the authors that partial KIT inhibition was still achieved but not enough to suppress all the KIT resistant mutations. Our study reported good relative dose intensity in both arms (94% in Arm A; 100% for imatinib and 86% for regorafenib in Arm B) but pharmacokinetic analysis of the blood samples collected is awaited to see if drugs levels correlate with clinical endpoints in Arm B.

Treatment-related toxicity with imatinib and regorafenib was similar to that expected for either agent alone. The most common grade 3 or 4 toxicity was anemia and diarrhea in Arm A and palmar-plantar erythrodysesthesia syndrome and hypertension in Arm B. Toxicity was higher in the alternating arm compared to single-agent imatinib affecting the tolerability of this regimen with many dose reductions and discontinuation of drug administration due to side effects. There were no drug-related deaths in the imatinib-only arm and one death due to myocardial infarction in the alternating arm.

The treatment paradigm for GIST is continuously evolving. There are now three further options for GIST: avapritinib, ripretinib and pimitespib. Updated results from the phase I NAVIGATOR study reported 84% ORR in patients with the rare D842V and other exon 18 mutations and avapritinib is now considered first-line therapy in the uncommon patients with metastatic GIST with a *PDGFRA* D842V mutation [23]. Ripretinib was compared with placebo in the phase III INVICTUS trial in patients with advanced GIST who had received three or more lines of therapy [24]. They reported a PFS of 6.3 vs. 1.0 months and an OS of 15.1 vs. 6.6 months in the ripretinib group, respectively, compared to placebo, but ripretinib has not been evaluated as the first-line treatment for advanced GIST. Ripretinib has also been compared against sunitinib following imatinib failure in advanced GIST in the INTRIGUE study [25]. This did not show a meaningful difference in the median progression free survivals. The CHAPTER-GIST-301 phase III trial of the heat shock protein 90 (HSP90) inhibitor pimitespib demonstrated improved PFS of 2.8 months versus 1.4 months and cross over adjusted overall survival compared to placebo in fourth line therapy after imatinib, sunitinib and regorafenib [26].

The ALTGIST trial was a well conducted international randomized study which recruited patients from 11 countries thus reducing the risk of bias and improving generalizability of the outcomes. The study adopted RECIST 1.1 standardized criteria in tumor response measurement which were centrally corroborated to improve the validity of the data. The premature discontinuation of the study led to a smaller sample size than originally planned could mean that a moderate treatment effect could be missed. However there were no signals seen that would warrant further investigation of this strategy.

In summary there was no meaningful difference in the primary endpoint of OTR and in PFS between the groups in this final analysis of AGITG ALT-GIST. The difference in the Kaplan-Meier estimate of OS and time to treatment failure at 12 months were also not significant. Post-hoc analyses to the time of last available follow-up found no differences in duration of response or PFS and OS at four years between the study arms. No new or unexpected toxicity was observed, with increased toxicity seen, as expected, in the alternating arm. This alternating schedule does not warrant further study. We conclude that clinicians should continue the current standard of first-line single-agent continuous imatinib until disease progression on highest tolerable dose.

## Supplementary information

Suppplemental appendix

Supplementary Figure 1

Supplementary Figure 2

ALTGIST Protocol V1.0

ALT GIST Protoocl V2.0

ALT GIST Protocol V3.0

ALT GIST HREC notification.

ALT GIST Protocol V4.0

Related Manuscript File

## Data Availability

De-identified individual participant data collected during the AGITG ALT-GIST trial will be shared after article publication with no end date. These data will be available to researchers who provide a methodologically sound proposal for the purposes of achieving specific aims outlined in that proposal. Proposals should be directed to the Clinical Trials Project Manager via email to altgist.study@sydney.edu.au and will be reviewed by the AGITG ALT-GIST study management committee. Requests to access data to undertake hypothesis-driven research will not be unreasonably withheld. To gain access, data requesters will need to sign a data access agreement and to confirm that data will only be used for the agreed purpose for which access was granted.
