# Supplementary material for: Imatinib alternating with regorafenib compared to imatinib alone for the first-line treatment of advanced gastrointestinal stromal tumor: The AGITG ALT-GIST intergroup randomized phase II trial
Source: Br J Cancer. 2025 Mar 25;132(10):897–904. doi: 10.1038/s41416-025-02983-w (PMC12081743; doi:10.1038/s41416-025-02983-w)
Supplement: Supplementary file 1 — Suppplemental appendix [file 41416_2025_2983_MOESM1_ESM.docx]

**Supplemental Appendix**

# Patient Enrolment by Study Centre and Principal Investigator

| **Study Centre** | **Country** | **Principal investigator** | **Patients enrolled** |
| --- | --- | --- | --- |
| Institut Bergonie | France | Antoine Italiano | 10 |
| Netherlands Cancer Institute -Antoni Van Leeuwenhoek | Netherlands | Neeltje Steeghs | 9 |
| The Norwegian Radium Hospital | Norway | Kjetil Boye | 8 |
| Border Medical Oncology | Australia | Christopher Steer | 6 |
| Canberra Hospital | Australia | Desmond Yip | 6 |
| Lund University Hospital | Sweden | Mikael Eriksson | 5 |
| Centre Leon Berard | France | Jean-Yves Blay | 5 |
| Institute Gustave Roussy | France | Axel Le Cesne | 4 |
| NCCS Singapore | Singapore | Dr Nagavalli Somasundaram | 3 |
| Sahlgrenska University Hospital, Gothenburg | Sweden | Jon Kindblom | 3 |
| Instituto Oncologico Veneto | Italy | Antonella Brunello | 3 |
| Peninsula and South East Oncology | Australia | Vinod Ganju | 2 |
| Bankstown Lidcombe Hospital | Australia | Ray Asghari | 2 |
| UCL Hospital | United Kingdom | Palma Dileo | 2 |
| Helsinki University Hospital, Helsinki | Finland | Heikki Joensuu | 2 |
| Princess Alexandra Hospital | Australia | Dr Vladimir Andelkovic | 1 |
| Prince of Wales | Australia | David Goldstein | 1 |
| Haukeland University Hospital, Bergen | Norway | Nina Jebsen | 1 |
| St James University Hospital Leeds | United Kingdom | Maria Marples | 1 |
| Royal Marsden Hospital (London) | United Kingdom | Charlotte Benson | 1 |
| National Cancer Institute - Slovakia | Slovakia | Jozef Sufliarsky | 1 |
| ICO L'Hospitalet - Hospital Duran i Reynals (Institut Catala D'Oncologia) | Spain | Xavier Garcia del Muro Solans | 1 |
| Leiden University Medical Center | Netherlands | H. Gelderblom | 1 |
| Royal Hobart Hospital | Australia | Ian Byard | 0 |
| Sir Charles Gairdner Hospital | Australia | Guy van Hazel | 0 |
| Ashford Cancer Centre Research | Australia | Dusan Kotasek | 0 |
| Flinders Medical Centre | Australia | Chris Karapetis | 0 |
| Tweed Hospital | Australia | Sumit Lumba | 0 |
| Calvary Mater Newcastle | Australia | Stephen Ackland | 0 |
| Lake Macquarie Private Hospital | Australia | Stephen Ackland | 0 |
| Nottingham University Hospitals NHS Trust - Nottingham City Hospital | United Kingdom | Ivo Hennig | 0 |
| University Hospital Birmingham – Queen Elizabeth Hospital | United Kingdom | Jennifer Sherriff | 0 |
| Beatson West of Scotland Cancer Centre | United Kingdom | Jeff White | 0 |
| Tampere University Hospital | Finland | Kaisa Sunela | 0 |
| Oulu University Hospital | Finland | Raija Kallio | 0 |
| Centre Georges-Francois-Leclerc | France | Isambert Nicolas | 0 |
| University Medical Center Groningen | Netherlands | A. Reyners | 0 |
|  |  | **Total** | **78** |

#

# International Trial Management Group

| **Name** | **Institution** | **Patients enrolled** | **Country** |
| --- | --- | --- | --- |
| Prof Heikki Joensuu | Helsinki University Hospital/ Scandinavian Sarcoma Group (SSG) | 2 | Finland |
| A/Prof Mikael Eriksson | Lund University Hospital/ SSG | 5 | Sweden |
| Prof Desmond Yip | Canberra Hospital | 6 | Australia |
| Prof Jean-Yves Blay | Centre Leon Berard; European Organisation for Research and Treatment of Cancer (EORTC) | 5 | France |
| Prof John Zalcberg | Monash University | - | Australia |
| Sandrine Marreaud | EORTC | - | Belgium |
| Laura De Meulemeester | EORTC | - | Belgium |
| Ann Marinus | EORTC | - | Belgium |
| Prof John Simes | NHMRC CTC, University of Sydney | - | Australia |
| Prof Val Gebski | NHMRC CTC, University of Sydney | - | Australia |
| David Espinoza | NHMRC CTC, University of Sydney | - | Australia |
| Ailsa Langford | NHMRC CTC, University of Sydney | - | Australia |
| Dr Sonia Yip | NHMRC CTC, University of Sydney | - | Australia |
| A/Prof Katrin Sjoquist | NHMRC CTC, University of Sydney | - | Australia |
| Dr Derrick Siu | NHMRC CTC, University of Sydney | - | Australia |
| Russell Conley | Australasian Gastrointestinal Trials Group (AGITG) | - | Australia |

# Australian Trial Management Committee

| **Name** | **Site** | **Patients enrolled** | **Position** |
| --- | --- | --- | --- |
| Prof Desmond Yip | Canberra Hospital | 6 | Medical Oncologist |
| Prof David Goldstein | Prince of Wales | 1 | Medical Oncologist |
| Prof Chris Karapetis | Flinders Medical Centre | 0 | Medical Oncologist |
| A/Prof Dusan Kotasek* | Ashford Cancer Centre | 0 | Medical Oncologist *Retired |
| Prof Steve Ackland | Calvary Mater Newcastle/ Lake Macquarie Private Hospital | 0 | Medical Oncologist |
| A/Prof Craig Underhill | Border Medical Oncology | 6 | Medical Oncologist |
| Prof Jayesh Desai | Royal Melbourne Hospital | - | Medical Oncologist |
| Prof John Zalcberg | Monash University | - | Medical Oncologist |
| Prof John Simes | NHMRC CTC, University of Sydney | - | NHMRC CTC Clinical Lead |
| Prof Val Gebski | NHMRC CTC, University of Sydney | - | Statistician |
| Dr Sonia Yip | NHMRC CTC, University of Sydney | - | NHMRC CTC Translational Research Lead |
| Mr David Espinoza | NHMRC CTC, University of Sydney | - | Statistician |
| Ms Ailsa Langford | NHMRC CTC, University of Sydney | - | Clinical Trial Operations Lead |
| A/Prof Katrin Sjoquist | NHMRC CTC, University of Sydney | - | NHMRC CTC Clinical Research Fellow |
| Dr Derrick Siu | NHMRC CTC, University of Sydney | - | NHMRC CTC Clinical Research Fellow |
| Russell Conley (Invitee) | AGITG | - | AGITG, Chief Executive Officer |

# Clinical Trial Registration

Australia New Zealand Clinical Trial Registry (ANZCTR) registration ID: ACTRN12614000950662

ClinicalTrials.gov registration ID: NCT02365441

# Collaborators

Bayer Healthcare Pharmaceuticals Inc. – Provided funding for trial and provision of study drug Regorafenib.

AGITG – Study sponsor

NHMRC CTC, University of Sydney – Global coordinating centre

EORTC – European regional coordinating centre

SSG – Scandinavian regional coordinating centre

PCI Pharma Services (previously Pharmaceutical Packaging Professionals PPP) – Australian distributor of Regorafenib

# Australian Coordinating Centre contributors (NHMRC CTC, University of Sydney)

| **Name** | **Role on study** |
| --- | --- |
| Professor John Simes | Clinical Lead |
| Professor Val Gebski | Statistician |
| David Espinoza | Statistician |
| Dr Sonia Yip | Translational Research Lead |
| A/Prof Katrin Sjoquist | Senior Clinical Research Fellow |
| Dr Derrick Siu | Clinical Research Fellow |
| Dr Deborah Zhou | Clinical Research Fellow |
| Dr Danielle Ferraro | Clinical Research Fellow (previous) |
| Dr Zarka Samoon | Clinical Research Fellow (previous) |
| Ailsa Langford | Clinical Trial Operations Lead |
| Jenna Mitchell | Clinical Trial Operations Lead (previous) |
| Nicole Wong | Clinical Trial Operations Lead (previous) |
| Cheryl Friend | Clinical Trial Operations Lead (previous) |
| Martijn Oostendorp | Clinical Trial Operations Lead (previous) |
| Deepa Mathur | Trial Operations Coordinator |
| Sarah York | Trial Operations Coordinator (previous) |
| Lauren Fisher | Trial Operations Coordinator (previous) |
| Savita Iyer | Trial Operations Coordinator (previous) |
| Jaclyn Verghis | Trial Operations Coordinator (previous) |
| Vevian Evans | Clinical Data Manager (previous) |
| Marco Pin | Clinical Trials Assistant |
| Rose Estefanos | Clinical Trials Assistant (previous) |

# European Coordinating Centre contributors (EORTC)

| **Name** | **Role on study** |
| --- | --- |
| Sandrine Marreaud | Senior Clinical Research and Development Lead |
| Ionela Stanciu | Clinical Operations Manager |
| Laura De Meulemeester | Clinical Operations Manager (previous) |

# Scandinavian Coordinating Centre contributors (SSG)

| **Name** | **Role on study** |
| --- | --- |
| Prof Heikki Joensuu | Trial Coordinating Principal Investigator |
| A/Prof Mikael Eriksson | SSG Clinical Lead |
| Eva-Mari Olofsson | Clinical Trials manager |

# Protocol versions

Version 1.0, dated 3^rd^ July 2014

Version 2.0, dated 16^th^ January 2017 (not implemented)

Version 3.0, dated 1^st^ August 2017

Version 4.0, dated 5^th^ October 2021

**Supplementary figures**

Supplementary figure 1

Kaplan Meier curves for progression free (Panel A) and overall survival (Panel B) up until last follow up of May 18, 2023

Supplementary figure 2

Kaplan Meier duration of response curves up until last follow up May 18, 2023
