# Supplementary material for: Imatinib alternating with regorafenib compared to imatinib alone for the first-line treatment of advanced gastrointestinal stromal tumor: The AGITG ALT-GIST intergroup randomized phase II trial
Source: Br J Cancer. 2025 Mar 25;132(10):897–904. doi: 10.1038/s41416-025-02983-w (PMC12081743; doi:10.1038/s41416-025-02983-w)
Supplement: Supplementary file 2 — Supplementary Figure 1 [file 41416_2025_2983_MOESM2_ESM.docx]

**Supplementary figure 1**

Kaplan Meier curves for progression free (Panel A) and overall survival (Panel B) up until last follow up of May 18, 2023

**
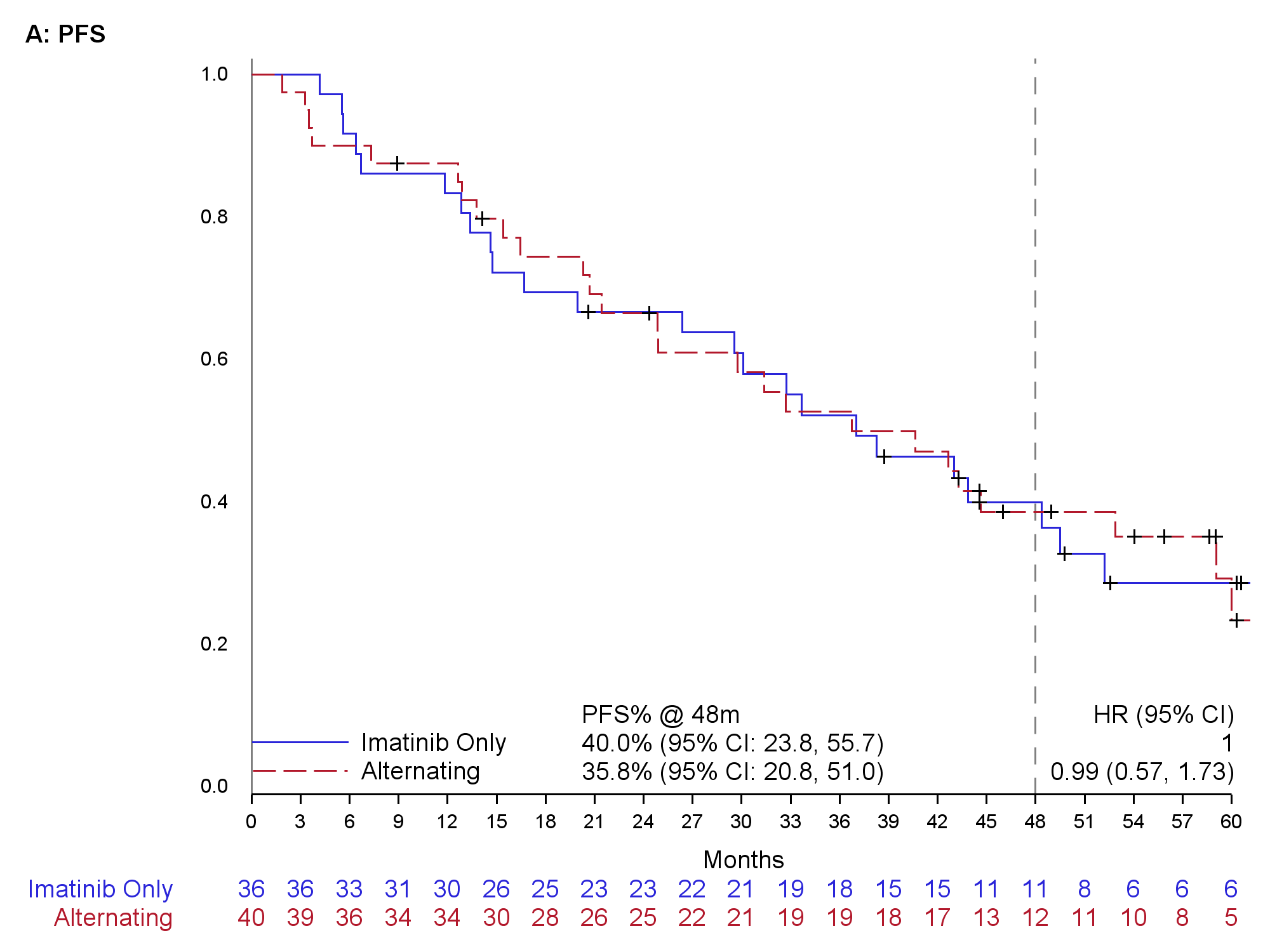
**

**
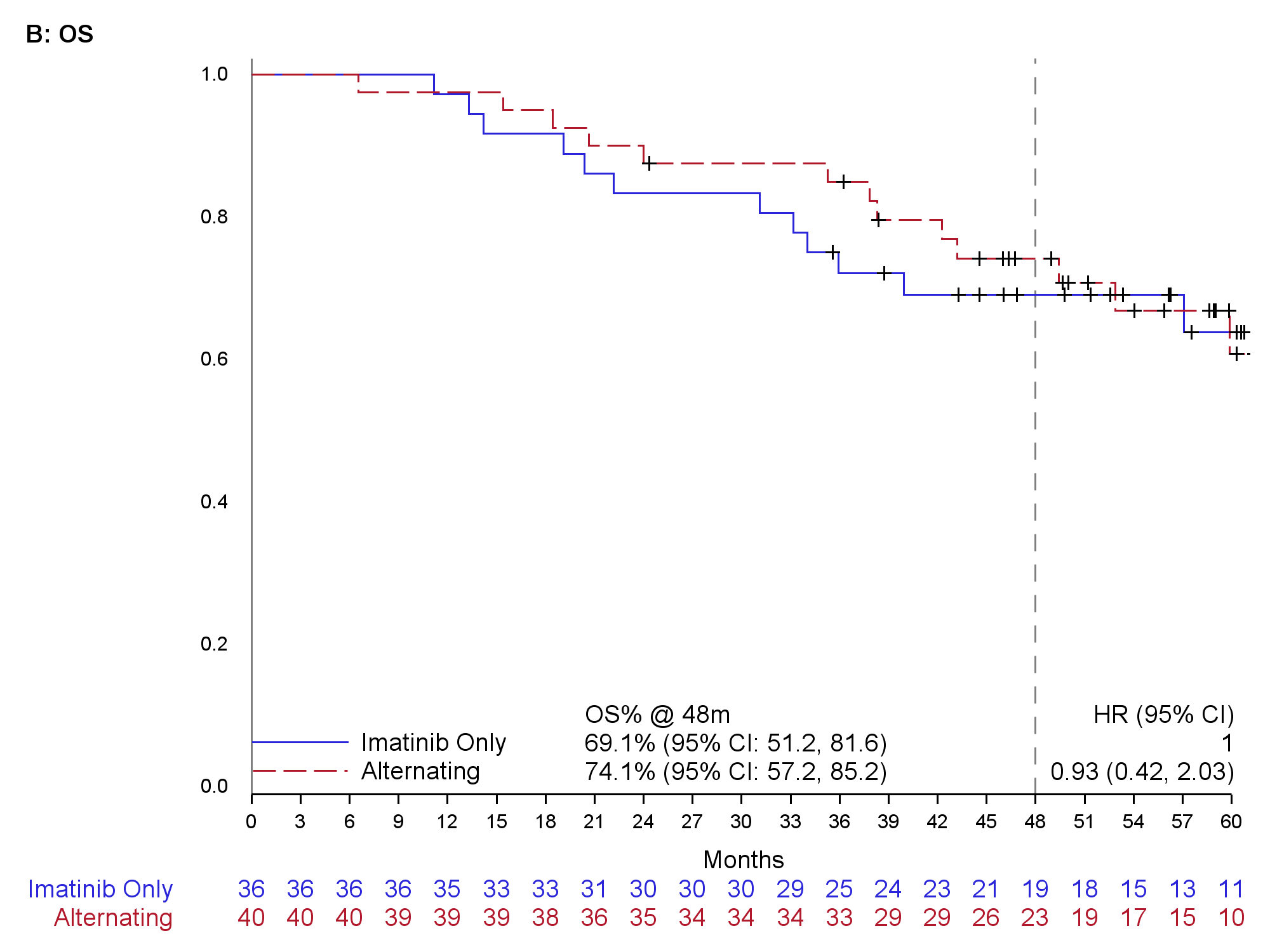
**
