# Supplementary figures and images for: Imatinib alternating with regorafenib compared to imatinib alone for the first-line treatment of advanced gastrointestinal stromal tumor: The AGITG ALT-GIST intergroup randomized phase II trial
Source: Br J Cancer. 2025 Mar 25;132(10):897–904. doi: 10.1038/s41416-025-02983-w (PMC12081743; doi:10.1038/s41416-025-02983-w)

**Supplementary figure 2**

Kaplan Meier duration of response curves up until last follow up May 18, 2023

**
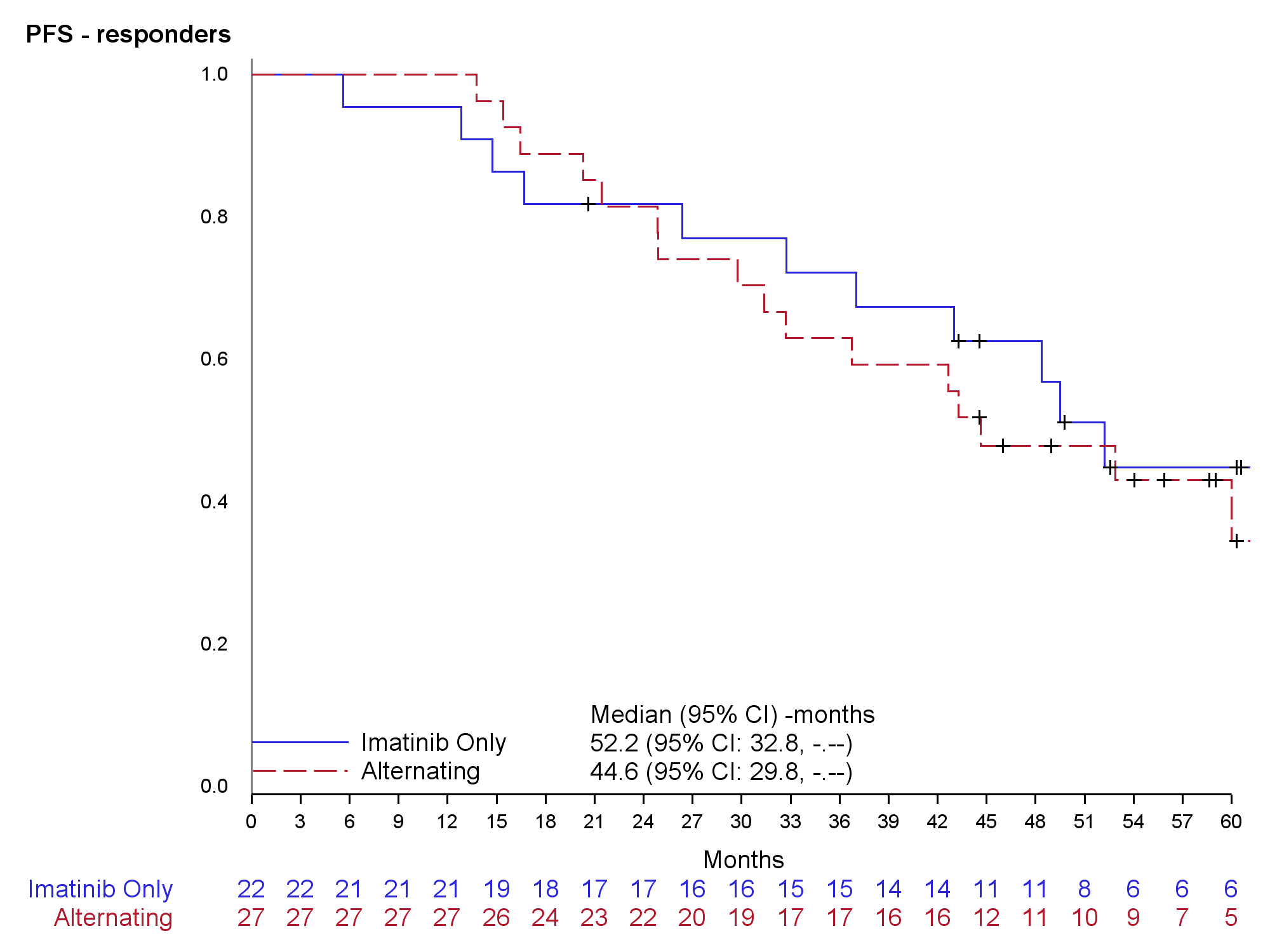
**

Supplement: Supplementary file 3 — Supplementary Figure 2 [file 41416_2025_2983_MOESM3_ESM.docx]
