# Supplementary material for: Imatinib alternating with regorafenib compared to imatinib alone for the first-line treatment of advanced gastrointestinal stromal tumor: The AGITG ALT-GIST intergroup randomized phase II trial
Source: Br J Cancer. 2025 Mar 25;132(10):897–904. doi: 10.1038/s41416-025-02983-w (PMC12081743; doi:10.1038/s41416-025-02983-w)
Supplement: Supplementary file 5 — ALT GIST Protoocl V2.0 [file 41416_2025_2983_MOESM5_ESM.pdf]

ALT GIST

A randomised phase II trial of imatinib alternating with regorafenib compared to imatinib alone for the first line treatment of advanced gastrointestinal stromal tumour (GIST)

NHMRC CTC Protocol number CTC 0122/AGITG AG1013GST

International Study Chair: Professor Heikki Joensuu
ANZ Study Chair: Professor Desmond Yip

Protocol version. 2.0, 16 Jan 2017

International Sponsor: Australasian Gastro-Intestinal Trials Group (AGITG)
119-143 Missenden Road
Camperdown NSW 2050

This study is collaboration between the AGITG, NHMRC Clinical Trials Centre (NHMRC CTC), European Organisation for Research and Treatment of Cancer (EORTC) and Scandinavian Sarcoma Group (SSG).

ANZ Coordinating and International Data Centre:

NHMRC Clinical Trials Centre
92-94 Parramatta Road
Camperdown NSW 2050
Telephone: 61-2-9562-5000
Fax: 61-2-9565-1863
Email: ALTGIST@ctc.usyd.edu.au

CONFIDENTIAL

©NHMRC Clinical Trials Centre

Senior Statistician Professor Val Gebski

CTC Clinical Lead Professor John Simes

Region	Coordinating Centre	Clinical Lead	Coordinating Centre Lead
ANZ and Asia	NHMRC CTC	Prof. Desmond Yip	Ms Cheryl Friend
Europe	EORTC	Prof Jean Yves Blay	Mr Ward Sents
Scandinavia	SSG	Prof Heikki Joensuu	Dr Mikael Eriksson

Protocol Development Working Party

The following individuals also contributed to the design and development of this protocol:

Name	Position	Organisation
Dr Wendy Hague	Director of Clinical Trials	NHMRC CTC
Dr Danielle Ferraro	Research Fellow – AGITG trials	NHMRC CTC
Ms Nicole Wong	Associate Oncology Program Manager	NHMRC CTC
Dr Sonia Yip	Translational Research Fellow	NHMRC CTC

 ©NHMRC Clinical Trials Centre

ALT GIST

Table of Contents

LIST OF ABBREVIATIONS 5
SYNOPSIS AND SCHEMA 6
1. BACKGROUND 10
2. REGORAFENIB 11
3. AIM AND OBJECTIVES 11
4. DESIGN 12
5. SUBJECT POPULATION 12
5.1. Target Population 12
5.2. Inclusion criteria 12
5.3. Exclusion criteria 13
6. STUDY ENROLMENT 14
6.1. Screening 14
6.2. Randomisation 14
7. TREATMENT PLAN 14
7.1. Administration of study treatments 14
7.2. Dose modifications 15
7.2.1. Dose levels 16
7.2.2. Treatment-Emergent Hypertension 18
7.2.3. Treatment Related Hepatic Toxicity 20
7.3. Concomitant Medications/Treatments 21
7.3.1. Recommended 21
7.3.2. Permitted 22
7.3.3. Use with caution 22
7.3.4. Prohibited 23
7.3.5. Concomitant medication reporting 23
7.4. Treatment discontinuation 23
7.5. Subsequent treatment 23
8. ASSESSMENT PLAN 24
8.1. Schedule of assessments 24
8.2. Assessment phase definitions and special circumstances 27
8.2.1. Baseline 27
8.2.2. During treatment 27
8.2.3. End of treatment 28
8.2.4. 30 day safety assessment 28
8.2.5. Follow-up after treatment 28
8.2.6. After study is closed 28
9. OUTCOMES, ENDPOINTS AND OTHER MEASURES 28
9.1. Objective tumour response (complete or partial response) at or before 9 months 28
9.2. Progression free survival (disease progression or death) 29
9.3. Clinical benefit rate at 3 cycles (24 weeks) 29
9.4. Time to treatment failure 29
9.5. Adverse Events (worst grade according to NCI CTCAE v4.03) 29
9.6. Overall survival 29
9.7. Rate of patients having macroscopically complete removal of all residual disease by surgery 29
9.8. Change in PET imaging during washout period of regorafenib and imatinib in those taking part in the PET substudy 29
9.9. Biomarkers 30
10. SAFETY REPORTING 30
10.1. Definitions 30
10.2. Reporting of Serious Adverse Events (including SUSARs) 31
10.3. Pregnancy 32

11. CENTRAL REVIEW AND BIOSPECIMEN COLLECTION 32
11.1. Central Biospecimen Collection 32
11.2. Central Imaging Collection 32
12. TREATMENT INFORMATION 33
12.1. Description of Study Products 33
12.2. Supply of Study Product 33
12.3. Drug Accountability 33
13. STATISTICAL CONSIDERATIONS 33
13.1. Sample Size 33
13.2. Statistical Analysis 34
13.3. Compliance Assessment 34
14. STUDY ORGANISATION 34
14.1. Trial Management Committee(s) 34
14.2. Independent Safety and Data Monitoring Committee 35
15. ADMINISTRATIVE ASPECTS 35
15.1. Ethics and regulatory compliance 35
15.2. Confidentiality 35
15.3. Protocol amendments 35
15.4. Data Handling and Record Keeping 36
15.5. Audit and Inspection 36
15.6. Clinical Study Report 36
16. REFERENCES 37
17. LIST OF APPENDICES 38
Appendix 1. National Cancer Institute Common Terminology Criteria for Adverse Events (CTCAE) Version 4.03 39
Appendix 2. Response Evaluation Criteria in Solid Tumours (RECIST Version 1.1) 40

LIST OF ABBREVIATIONS

AE	Adverse Event
ALP	Alkaline phosphatase
ALT	Alanine aminotransferase
AST	Aspartate aminotransferase
BP	Blood Pressure
CBR	Clinical benefit rate
CTCAE	Common Terminology Criteria for Adverse Events
CR	Complete Response
CTC	NHMRC Clinical Trials Centre
DNA	Deoxyribonucleic acid
EGFR	Epidermal Growth Factor receptor
FDA	Food and Drug Administration
GIST	Gastrointestinal Stromal Tumour
GP	General Practitioner
HREC	Human Research Ethics Committee
HIV	Human immunodeficiency virus
IDSMC	Independent Data, Safety Monitoring Committee
INR	International Normalised Ratio
ISC	International Steering Committee
NYHA	New York Heart Association
OTRR	Objective tumour response rate
PET	Positron emission tomography
PDGFRA	Platelet-derived growth factor receptor alpha
PFS	Progression Free Survival
PD	Progressive Disease
PR	Partial Response
SAE	Serious adverse event
SD	Stable Disease
SUSAR	Suspected Unexpected Serious Adverse Reaction
SUV	Standardised uptake values
TCM	Traditional Chinese medicine
TFT	Thyroid function test
TGA	Therapeutic Goods Administration
TMC	Trial Management Committee
TKI	Tyrosine Kinase inhibitor
ULN	Upper limit of normal

 ©NHMRC Clinical Trials Centre

ALT GIST

SYNOPSIS AND SCHEMA

PROTOCOL SYNOPSIS

Background	Despite highly active current treatment for metastatic gastrointestinal stromal tumour (GIST) with the use of imatinib, most people will ultimately relapse and die of multifocal metastatic disease. Using an alternating regimen of imatinib and regorafenib with brief drug free intervals may allow tumour stem cells to re-enter the cell cycle and become susceptible once more to drug therapy. Regorafenib, a multi-targeted tyrosine kinase inhibitor (TKI) with activity against angiogenic, stromal and oncogenic receptor tyrosine kinases, has demonstrated activity in the treatment of GIST and is FDA approved for third line therapy of advanced GIST.
General aim	To determine if an alternating regimen of imatinib and regorafenib has sufficient activity and safety to warrant further evaluation as a first line treatment for metastatic GIST.
Primary objective (endpoint)	▪ Objective tumour response (complete or partial response) as determined by RECIST v1.1 at or before 9 months from the time from either (i) randomization (if patients have not yet commenced treatment) or (ii) commencement of therapy (if patients are randomized during the first cycle of imatinib)
Secondary objectives (endpoints)	▪ Progression Free Survival<br>▪ Clinical benefit rate (SD + PR + CR) following 3 cycles (24 weeks) of treatment<br>▪ Time-to-treatment failure<br>▪ Safety/toxicity/tolerability<br>▪ Overall survival
Exploratory objectives (endpoints)	To explore the relationship between study endpoints and:<br>▪ Change in PET imaging during washout period of regorafenib and imatinib (in subset of participants at selected centres)<br>▪ Imatinib plasma levels in Arm A and in Arm B imatinib and regorafenib plasma levels.<br>▪ Circulating biomarkers as prognostic and/or predictive markers including, but not limited to:<br>o Circulating serum/plasma growth factor and cytokine levels (multiplex assay)<br>o KIT/PDGFRA mutations in circulating blood DNA (‘liquid biopsy’)<br>o DNA load<br>▪ Tumour tissue biomarkers including, but not limited to, proteins relating to KIT and PDGFR signalling and

angiogenesis
- Rate of patients having macroscopically complete removal of all residual disease by surgery

Design	Prospective, randomised, open label phase II trial, stratified by participating site, previous adjuvant therapy (prior vs none), and previous imatinib for metastatic disease for less than or equal to 25 days prior to randomisation.
Population	The target population is adults with histologically confirmed, measurable metastatic GIST, who have received no prior treatment for metastatic disease. Patients who are currently taking, and have not had longer than 25 days of continuous treatment immediately prior to randomisation with 400mg daily of imatinib are eligible to participate in this study.
Study treatments	Patients will be randomised to receive either:<br>Arm A – imatinib 400mg orally daily continuously (control arm); or<br>Arm B – alternating 28-day periods of imatinib 400mg orally daily for 21 to 25 days followed by a washout (drug free) period of 3 to 7 days, then regorafenib 160mg orally daily for 3 weeks followed by a 7 day washout (drug free) period.<br><br>Treatment will continue until disease progression or prohibitive adverse events as detailed in the protocol.
Assessments	▪ Clinic visits (including laboratory tests) will be required at baseline, prior to each cycle of treatment and at the end of study treatment visit. During Cycles 1, 2 and 3, clinic visits will occur prior to commencing each new agent (4 weekly) including blood tests for haematological, hepatic, thyroid and renal function.<br>▪ Additional liver function tests will be performed weekly during the first 2 cycles of regorafenib treatment for those patients in the alternating arm (Arm B) of the study.<br>▪ Imaging will be performed at baseline (within 21 days prior to randomisation) and every 8 weeks for the first year on trial, and then every 12 weeks until disease progression or death, timed from the randomisation date.<br>▪ Blood samples for biomarker studies will be collected at baseline. And in Arm B every 4 weeks for the first 2 cycles of treatment then at commencement of cycle 4 , 8 and disease progression. And in Arm A in cycle 1,2, 4, 8 and disease progression.<br>▪ Blood for drug (imatinib) plasma level- will be collected in Arm A cycle 1 week 4 and cycle 2 week 4. For patients on the alternating regimen (Arm B) blood will be collected

for drug (imatinib) level on cycle 1, on the last day of imatinib administration and at cycle 2 on last day of imatinib administration. Blood will be collected for drug (regorafenib) level on cycle 1 week 7 (on last day of regorafenib administration) and cycle 2 week 7 (on last day of regorafenib administration).

- For those involved in the PET sub study, 2 PET scans will be performed, one at the commencement of the drug washout period and one at the conclusion of the drug washout period in cycle 2 at the earliest or in a subsequent cycle (at the first opportunity after a complete cycle of treatment is given).

Statistical considerations

This is a non-comparative randomized phase II design of 60 evaluable patients allocated 2:1 (38 in the alternating group and 22 in the control), The primary outcome is the objective tumour response rate by 9 months. Based on accrual of 38 evaluable patients in the alternating group, the table below, based on the charts of Mehta and Cain, give the minimum number of responses needed to be observed to be consistent with different response rate.

	True response rate (%)			
	30	40	50	60
Number of responses	≥ 7	≥ 10	≥ 13	≥ 17

These limits are based on 95% lower confidence intervals for the respective response rates.

 ©NHMRC Clinical Trials Centre

Study Schema

Eligibility

Previously untreated patients with metastatic GIST#

Stratification

- Prior adjuvant therapy (Y/N)
- Hospital Site
- Commenced Imatinib for metastatic disease ≤ 25 days prior (Y/N)*

R

Imatinib 400mg daily ongoing until progression.

Imatinib 400mg for 21 to 25 days with a 3-7 day washout period followed by Regorafenib 160mg for 21 days followed by a 7 day washout period. Ongoing treatment until progression.

Endpoints

- Objective tumour response (Primary)
- Progression free survival
- Clinical benefit rate
- Time-to-treatment failure
- Safety/Toxicity/Tolerability
- Overall Survival

NOTE:

Cycles are 8 weeks (56 days) in length for both arms.

Not suitable for surgery with curative intent

* Patients who have already commenced imatinib may be randomized at any time in the first 25 days of treatment. For these patients, the number of days that imatinib has already been taken for will be **included** in the treatment period (ie this prior treatment will count towards the total days of imatinib in cycle 1).

©NHMRC Clinical Trials Centre

1. BACKGROUND

While modern treatment of metastatic gastrointestinal stromal tumour (GIST) with imatinib is highly active with clinical benefit rates of over 80%, most people will ultimately relapse. Two large pivotal randomized trials of 400mg versus 800mg of imatinib conducted in Europe/Scandinavia/Australasia and North America respectively (1, 2), demonstrated the median progression free survival (PFS) was approximately two years. However more recent data suggests that the median PFS is longer than this in patient populations with a small tumour burden at the time of imatinib initiation (3). Nevertheless clinical experience and longer follow up has confirmed that relatively few people are cured, with most people eventually dying of multifocal disease progression.

In these circumstances, tumours may express varying mechanisms of resistance to imatinib (4) as well as second generation tyrosine-kinase inhibitors (TKI), due either to the acquisition of secondary mutations in *KIT* (5) or potentially the selection of pre-existing drug-resistant clones (6). The emergence of resistance to imatinib occurs despite initial response rates of well over 70% in the more sensitive tumours carrying mutations in exon 11 of *KIT.*

Given the exquisite sensitivity of GIST cells to imatinib *in vitro* (7), and the dramatic clinical effect of imatinib administered to people in the first-line setting (8), particularly in tumours carrying mutations in *KIT* exon 11 (representing approximately 65% of all patients), it is unclear why complete responses are comparatively rare and the cure rate so low, with the vast majority of people dying with advanced disease.

A potential explanation for this observation relates to the presence of GIST stem cells – Bardsley et al have identified progenitors for Interstitial Cells of Cajal (ICC) which act as stem cells for GIST. These cells expressed Cd44 (in a similar manner to some other cancer stem cells) and Cd34, and were capable of self-renewal and differentiation. They formed malignant tumours that expressed GIST markers when spontaneously transformed stem cells were implanted in nude mice. These stem cells had innately low *KIT* expression, and were thus resistant to in-vitro blockade by *KIT* inhibitors. The progeny cells however rely on KIT signalling for their survival and differentiation (9). We hypothesise that by eliminating the selective pressure of continued pharmacologic therapy - which encourages the proliferation of resistant clones - by temporarily discontinuing treatment ('washout period'), and using a second TKI such as regorafenib, with established activity in GIST (10), response rates and survival times may improve for patients with this malignancy.

Incorporating a brief TKI- free interval (washout period) into the treatment schedule may allow dormant cells to become active again, and quiescent stem cells responding to higher than pre-treatment levels of stem cell factor (a ligand for the extracellular domain of *KIT*, known to increase during treatment for metastatic GIST (11)) may also re-enter the cell cycle. This cell re-activation should result in even greater tumour apoptosis with the subsequent recommencement of TKI therapy.

Tumour cells harbouring secondary mutations that expand predominantly because of this selective pressure may also regress during a brief period free of TKI exposure, and become more susceptible to therapy.

Given the above rationale, it is predicted that with the addition of a second targeted agent, regorafenib (a drug with very substantial activity, at least in the third-line setting in GIST (10)) after a drug-free

period, residual tumour cells may be eradicated and some partial responders will be converted into complete responders.

2. REGORAFENIB

Regorafenib (12) is a TKI with inhibitory activity against VEGFR1-3, bFGFR, PDGFRA, Tie 2, BRAF, CRET RaFi p38 MAPK and KIT with an IC50 *in vitro* of approximately 10nM. In a randomised, placebo controlled phase 3 trial in 199 patients who had failed at least 2 previous lines of therapy for GIST, there was a statistically significant reduction in the risk of progression in the regorafenib arm compared to the placebo arm (median PFS 4.8 vs. 0.9 months, HR 0.27)(13). During the double blind phase of the same trial, the most common grade 3 or higher adverse events recorded in the regorafenib arm were hypertension (23%), hand-foot syndrome (20%), and diarrhoea (5%). Dose modifications were required more frequently in the regorafenib arm compared to the placebo arm, but the rate of permanent treatment discontinuation due to adverse events in the regorafenib arm (6%) was similar to that in the placebo group (8%).

These strategies for improving response to therapy (treatment-free periods and addition of a second active agent) are incorporated in the design of this pragmatic trial, which aims to increase the PFS rate and ultimately increase the cure rate for advanced GIST.

The purpose of this study is to determine if an alternating regimen of imatinib and regorafenib has sufficient activity and safety to warrant further evaluation as a first line treatment for metastatic GIST. If this strategy proves beneficial it has the potential to change treatment in this setting.

3. AIM AND OBJECTIVES

General aim	To determine if an alternating regimen of imatinib and regorafenib has sufficient activity and safety to warrant further evaluation as a first line treatment for metastatic GIST.
Primary objective (endpoint)	▪ Objective tumour response by 9 months as determined by RECIST v1.1. from either the date of (i) randomization (if patients have not yet commenced treatment) or (ii) commencement of therapy (if patients are randomized during the first cycle of imatinib)
Secondary objectives (endpoints)	▪ PFS as calculated from either the date of (i) randomization (if patients have not yet commenced treatment) or (ii) commencement of therapy (if patients are randomized during the first cycle of imatinib)Clinical benefit rate (SD + PR + CR) following 3 cycles (24 weeks) of treatment<br>▪ Time to treatment failure<br>▪ Safety/toxicity/tolerability<br>▪ Overall survival
Tertiary and correlative objectives	To explore the relationship between study endpoints and:<br>▪ Change in PET imaging during washout period of regorafenib and imatinib (in subset of participants at

selected centres)

- Imatinib plasma levels in Arm A and imatinib and regorafenib plasma levels in Arm B.
- Circulating biomarkers as prognostic and/or predictive markers including but not limited to:
    - Circulating serum/plasma growth factor and cytokine levels (multiplex assay)
    - KIT/PDGFRA mutations in circulating blood DNA ('liquid biopsy')
    - DNA load
- Tumour tissue biomarkers including, but not limited to, proteins relating to KIT and PDGFR signalling and angiogenesis.
- Rate of patients having macroscopically complete removal of all residual disease by surgery

4. DESIGN

Prospective, randomised, open label phase II trial, with randomisation 2:1 by minimisation and stratification by site, receipt of previous adjuvant therapy (prior vs none), and receipt of imatinib for metastatic disease for less than or equal to 25 days.

5. SUBJECT POPULATION

Patients must meet all of the inclusion criteria and none of the exclusion criteria to be eligible for this trial. There will be no exceptions made to these eligibility requirements at the time of randomisation. All enquiries about eligibility should be addressed by contacting the regional site coordinating centre prior to randomisation.

5.1. Target Population

Eligible participants with metastatic GIST (not suitable for surgery with curative intent), with no previous treatment for metastatic disease.

5.2. Inclusion criteria

1. Adults (over 18 yrs) with histologically confirmed GIST. In CD-117-negative cases, DOG-1 must be positive or a *KIT*/*PDGFRA* mutation must be present.
2. Unresectable, metastatic disease.
3. No prior TKI for metastatic disease, with the exception of those patients who have had up to and including 25 days of uninterrupted treatment on 400mg daily of imatinib.
4. Imatinib therapy given as an adjuvant treatment and completed at least 3 months prior to entry into this trial is permitted. Patients who have progression of GIST while on adjuvant therapy are not eligible for this trial.
5. ECOG performance status 0-2.
6. Measurable disease by RECIST version 1.1. (Note: Participants with only peritoneal disease will be eligible only if they have lesions measurable in two dimensions and have at least 1 lesion which is ≥ 2 cm in size).

7. Adequate bone marrow function (Haemoglobin ≥ 9.0g/dL, platelet count ≥ 100 x 10^9/L, and absolute neutrophil count ≥ 1.5 x 10^9/L).
8. Adequate liver function (Serum total bilirubin ≤1.5 x ULN, INR ≤ 1.5, and ALT, AST, ALP ≤2.5 x ULN (≤ 5 x ULN for participants with liver metastases).
9. Adequate renal function (Creatinine clearance > 50ml/min) based on either the Cockcroft Gault formula, 24 hour urine or Glomerular Filtration Rate (GFR scan); and serum creatinine ≤ 1.5 x ULN.
10. Tumour tissue available for central review.
11. Willing and able to comply with all study requirements, including treatment timing and/or nature of required assessments.
12. Study treatment both planned and able to start within 14 days of randomisation.
13. Signed, written informed consent.

5.3. Exclusion criteria

1. Concurrent GI illness which may prevent absorption of imatinib or regorafenib – please note that prior gastrectomy or bowel resection does not exclude patients from this study.
2. Use of other investigational drugs within 4 weeks prior to enrolment.
3. Known sensitivity to any of the study drugs, study drug classes, or excipients in the formulation.
4. Participants receiving therapeutic doses of warfarin.
5. Presence of brain metastases.
6. The presence of known *PDGFRA* D842V mutation or other mutation known to cause imatinib resistance.
7. Inability to swallow tablets.
8. Arterial thrombotic or ischaemic events, such as cerebrovascular accident or pulmonary embolism within 6 months prior to randomisation; or major venous thrombotic events requiring use of an anticoagulant such as warfarin within 6 months prior to randomisation.
9. Poorly controlled hypertension (systolic blood pressure > 140 mmHg or diastolic pressure > 90 mmHg despite optimal medical management).
10. Major surgical procedure, open biopsy, or significant traumatic injury within 28 days prior to randomisation, or non healing wound, ulcer or fracture.
11. Congestive cardiac failure (NYHA ≥ grade 2), unstable angina or new onset angina within the previous 3 months, or AMI within the previous 6 months. Cardiac arrhythmias requiring anti-arrhythmic therapy (beta blockers or digoxin are permitted).
12. Haemorrhage or bleeding event ≥ Grade 3 according to CTCAE v4.03 within 4 weeks prior to randomisation.
13. Ongoing infection of > Grade 2 according to CTCAE v4.03.
14. Active hepatitis B or C or HIV, or chronic hepatitis B or C requiring treatment with antiviral therapy. Testing for these is not mandatory unless clinically indicated.
15. Interstitial lung disease with ongoing signs and symptoms.
16. Persistent proteinuria of ≥ Grade 3 (>3.5g/24 hours) according to CTCAE v4.03
17. Other significant medical or psychiatric condition judged by the investigator to interfere with protocol requirements.
18. Use of biological response modifiers such as granulocyte colony stimulating factor (G-CSF), within 3 weeks prior to randomisation.
19. Patients taking strong cytochrome P (CYP) CYP3A4 inhibitors (eg clarithromycin, indinavir, itraconazole, ketoconazole, nefazodone, nelfinavir, posaconazole, ritonavir, saquinovir,

 ©NHMRC Clinical Trials Centre

telithromycin, voriconazole) or strong CYP3A4 inducers (eg carbamazepine, phenobarbitol, phenytoin, rifampicin, St John's wort).

20. History of another malignancy within 5 years prior to registration. Patients with a past history of adequately treated carcinoma-in-situ, basal cell carcinoma of the skin, squamous cell carcinoma of the skin, or superficial transitional cell carcinoma of the bladder are eligible. Patients with a history of other malignancies are eligible if they have been continuously disease free for at least 5 years after definitive primary treatment.
21. Pregnancy, lactation, or inadequate contraception. Women must be post menopausal, infertile, or use a reliable means of contraception. Women of childbearing potential must have a negative pregnancy test done within 7 days prior to registration. Women of childbearing potential and men must agree to use adequate contraception before entering the trial until at least 8 weeks after the last study drug administration.

6. STUDY ENROLMENT

6.1. Screening

Written informed consent must be signed and dated by the participant, and signed and dated by the Investigator, prior to any study-specific screening investigations being performed. In CD117 negative patients, entry to the study is conditional on the outcome of the DOG-1 expression at immunohistochemistry or *KIT/PDGFRA* mutational status.

6.2. Randomisation

Randomisation will be done according to the instructions in the Study Manual. Participants will be randomly assigned electronically by a computerised system.

Randomisation should be done only after all screening assessments have been performed and the responsible investigator has verified the participant's eligibility.

Randomisation occurs once all the inclusion criteria has been met.

Once the randomisation processes have been completed as per the instructions in the Study Manual, the patient will be assigned a subject study number, a treatment arm, and confirmation of successful randomisation is provided to the site.

Individuals may only be randomised once in this trial.

7. TREATMENT PLAN

7.1. Administration of study treatments

Participants randomised to standard therapy (Arm A) will receive 400mg (1 x 400mg tablet) orally daily of imatinib, with no washout periods.

Those randomised to the experimental alternating arm (Arm B) will receive:

Imatinib 400mg (1 x 400mg tablet) orally daily for 21 to 25 days, then a 3 - 7 day washout period (no drug). A 3 day washout period is mandated, but up to 7 days break is allowed to maximise tolerability of the regimen, at the discretion of the site investigator. In total, the combined number of days taking imatinib plus the washout period must equal 28 days. This is followed by regorafenib 160 mg (4 x 40mg tablets) orally daily for 21 days, then a 7 day washout period.

This combination of imatinib, regorafenib and intervening washout periods will constitute 1 cycle of study treatment (imatinib plus washout period 28 days in total; regorafenib plus washout period 28 days in total; cycle duration 8 weeks (56 days) in total). See diagram below.

It is recommended that regorafenib should be taken orally at the same time each day (preferably after breakfast) and the tablets should be swallowed whole with water after a light low fat meal. The prescribed dose of imatinib should be taken orally, with a meal and a large glass of water to minimise the risk of gastrointestinal disturbances

Missed or vomited tablets cannot be compensated for by treatment at a later date and/or time.

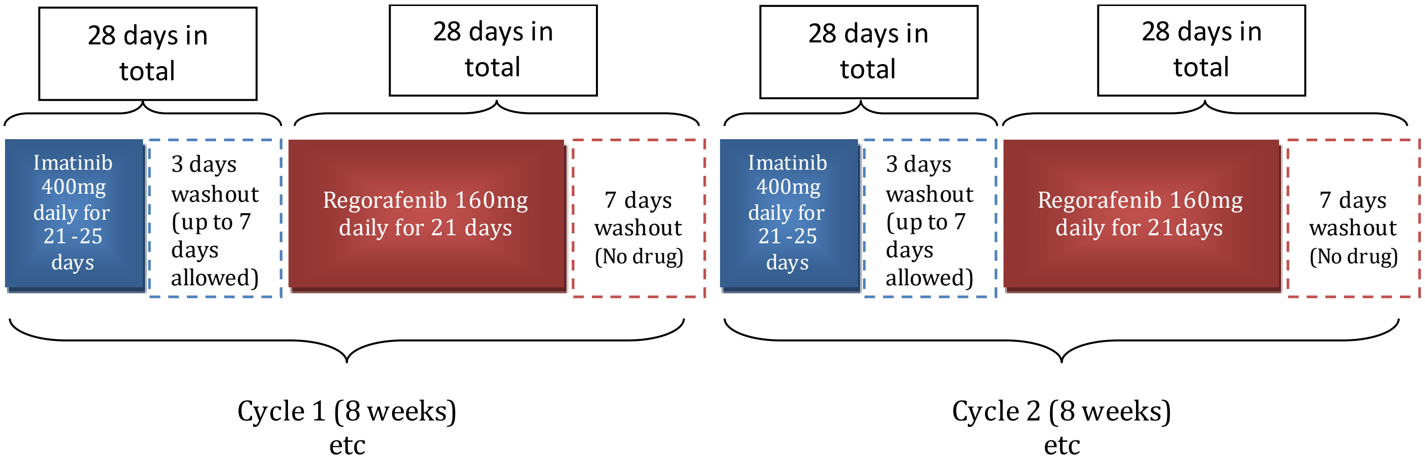

Even in participants with a complete response, treatment will continue until progressive disease (PD) or prohibitive adverse events are documented. Participants who cease treatment after receiving at least 2 years of therapy from the time of commencing treatment will not be considered to have failed treatment for toxicity reasons. Surgery will be permitted for participants whose cancer becomes operable during trial treatment at the discretion of the treating team, and participants will be able to continue on study treatment and will continue to be followed for progression free survival post operatively. For patients undergoing surgery, regorafenib should be ceased at least 2 weeks prior to surgery, and recommenced on the basis of clinical judgement. During this period, imatinib could be administered until surgery and for the first 2 weeks after surgery regardless of the study Arm allocation,

Treatment after progression will be according to clinician preference and local institutional guidelines. Crossover from either arm to single agent regorafenib will not be permitted as part of the study.

7.2. Dose modifications

Dose escalation:

Regorafenib dosages will not be increased above 160mg daily.

Dose escalation of imatinib up to 800mg daily will be allowed in both arms for those participants with an identified *KIT* exon 9 mutation. It is recommended in this situation that the patient be escalated first to 600mg for at least 2 weeks prior to further escalation to 800mg. The timing of dose escalation for *KIT* exon 9 mutation patients during the trial is at the discretion of the site investigator.

Dose reductions and delays:

Instructions for treatment delays and dose modifications for adverse events are specified below. In general, treatment should be withheld during adverse events of severity G3-4, and not restarted until the adverse event has resolved to G0-1, at the investigator's discretion unless otherwise specified. Day 1 treatment may be delayed for a maximum of 28 days. If the adverse event has not resolved to G0-1 after this delay, then study treatment should be discontinued. Treatment should not be delayed or modified for alopecia of any grade.

Specified dose reductions apply to all subsequent doses of study drug. If a patient experiences several adverse events with differing recommendations, then the modification that results in the longest delay and lowest dose should be used.

Dose modification guidelines differ for each arm of the study (see below). For Arm A, dose modifications are provided for adverse events thought to be attributable to imatinib (Table 2). For Arm B, dose modifications are provided for adverse events specific to regorafenib (hand-foot syndrome, hypertension and hepatic dysfunction)- see Tables 3-5, and separately for general adverse events thought related to either regorafenib or imatinib (including nausea, haematologic abnormalities, fatigue) – see Table 5.

7.2.1. Dose levels

Table 1:

DRUG	Units	STARTING DOSE			LEVEL		
			-2	-1	0 START	+1	+2
Imatinib	mg	400	200	300	400	600[a]	800[a]
Regorafenib	mg	160	80	120	160	----	----

a. Only patients with *KIT* exon 9 mutations are permitted to have imatinib dose escalations above 400mg.

In Arm A (standard therapy arm), dose modifications for adverse events thought to be related to imatinib are shown below:

Table 2:

Toxicity	Grade 1	Grade 2	Grade 3	Grade 4
Non-haematologic	Continue at the same dose level	Continue at the same dose level	Withhold dose until toxicity is grade ≤1, then resume at same dose level. If event recurs, withhold dose until toxicity is grade ≤ 1 and reduce dose for next cycle by 1 level. Dose reduce by 1 further level for further grade 3 or higher toxicity.	Withhold dose until toxicity is grade ≤1, then reduce dose for next cycle by 1 level. Dose reduce by 1 further level for further grade 3 or higher toxicity.

Haematologic (excluding anaemia **)	Continue at the same dose level.	Continue at the same dose level	Withhold dose until neutrophils ≥1.0 and platelets ≥100, then resume treatment at the same dose level. If event recurs, withhold dose until neutrophils ≥1.0 and platelets ≥100, and reduce dose for next cycle by 1 level. Dose reduce by 1 further level for further grade 3 or higher toxicity.	Withhold dose until neutrophils ≥1.0 and platelets ≥100, then reduce dose for next cycle by 1 level. Dose reduce by 1 further level for further grade 3 or higher toxicity.

**No dose modifications are required for Grade 1–4 anaemia however transfusion is permitted.

In Arm B (alternating therapy arm) for events specifically related to regorafenib (skin toxicity, treatment emergent hypertension, and hepatic dysfunction), see tables below:

Dose modifications for skin toxicity including Hand-foot skin reaction.

Table 3:

**Skin Toxicity Grade**	**Occurrence**	**Dose Modification**[a]
Grade 1: Numbness, dysaesthesia, paraesthesia, tingling, painless swelling, erythema or discomfort of the hands or feet which does not disrupt the participant's normal activities.	Any	Maintain dose level and immediately institute supportive measures for symptomatic relief.
Grade 2: Painful erythema and swelling of the hands or feet and/or discomfort which affects the participant's normal activities.	1st occurrence	Decrease dose by one dose level and immediately institute supportive measures. If there is no improvement, interrupt therapy for a minimum of 7 days, until toxicity resolves to Grade 0-1[c].
	No improvement within 7 days or 2nd occurrence	Interrupt therapy until toxicity resolves to Grade 0-1. When resume treatment, decrease dose by one dose level[c].
	3rd occurrence	Interrupt therapy until toxicity resolves to Grade 0-1. When resume treatment, decrease dose by one additional dose level[b,c].
	4th occurrence	Discontinue study treatment permanently.
Grade 3: Moist desquamation, ulceration, blistering or severe pain of the hands or feet, or severe discomfort that causes the	1st occurrence	Institute support measures immediately. Interrupt therapy for a minimum of 7 days until toxicity

participant to be unable to work or perform activities of daily living.		resolves to Grade 0-1. When resume treatment, decrease dose by one dose level.
	2nd occurrence	Institute support measures immediately. Interrupt therapy for a minimum of 7 days until toxicity resolves to Grade 0-1. When resume treatment, decrease dose by one additional dose level [b].
	3rd occurrence	Discontinue study treatment permanently.

a. More conservative management is allowed if judged medically appropriate by the Investigator.
b. Participants requiring > 2 dose level reductions should discontinue protocol therapy permanently.
c. If toxicity returns to grade 0-1 after dose reduction, dose re-escalation is permitted at the discretion of the investigator.

If there is no recovery after a delay of 28 days, treatment will be discontinued permanently.

For participants who require a dose reduction for grade 2 or 3 rash or hand-foot skin reaction, the dose of study drug may be increased to the starting dose after one full cycle of therapy has been administered at the reduced dose without the reappearance of rash or hand-foot skin reaction > grade 1.

7.2.2. Treatment-Emergent Hypertension

The dose modification schedule for treatment-emergent hypertension during study drug dosing should be followed. Participants' Blood Pressure (BP) measurements will be monitored and appropriate treatment to effectively control hypertension is required.
(Table on next page).

©NHMRC Clinical Trials Centre

Table 4:

**NCI-CTCAE v4.03 Grade**	**Definition**	**Anti-Hypertensive Therapy**	**Regorafenib dosing**
Grade 2	Systolic BP 140-159 mmHg or diastolic BP 90-99 mmHg<br>OR<br>Symptomatic increase by > 20 mmHg (diastolic) if previously within normal limits	Treat with the aim to achieve diastolic BP ≤ 90 mmHg<br>- If BP previously within normal limits, start anti-hypertensive monotherapy<br>- If participant already on anti-hypertensive medication, titrate up the dose.	Continue regorafenib.<br>If symptomatic, hold regorafenib until symptoms resolve AND diastolic BP ≤ 90 mmHg [a].<br>When regorafenib is restarted, continue at the same dose level
Grade 3	Systolic BP ≥ 160 mmHg or diastolic BP ≥100 mmHg<br>OR<br>More than one anti-hypertensive medication or more intensive therapy than previously used indicated	Treat with the aim to achieve diastolic BP ≤ 90 mmHg<br>- Start anti-hypertensive medication<br>AND/OR<br>- Increase current anti-hypertensive medication<br>AND/OR<br>- Add additional anti-hypertensive medications.	Hold regorafenib until diastolic BP ≤ 90 mmHg, and if symptomatic, until symptoms resolve [a].<br>When regorafenib is restarted, continue at the same dose level<br>If BP is not controlled with the addition of new or more intensive therapy, reduce by 1 dose level [b].<br>If Grade 3 hypertension recurs despite dose reduction and antihypertensive therapy, reduce another dose level [c]
Grade 4	Life-threatening consequences (e.g. malignant hypertension, transient or permanent neurologic deficit, hypertensive crisis)		Discontinue study treatment permanently

a. Participants requiring a delay of 28 days will be permanently discontinued.
b. If blood pressure remains controlled for at least one full cycle, dose re-escalation is permitted at the Investigator's discretion.
c. Participants requiring > 2 dose level reductions will be permanently discontinued.

7.2.3. Treatment Related Hepatic Toxicity

For participants with observed worsening of serum liver tests considered related to study drug (i.e. where no alternative cause is evident, such as post-hepatic cholestasis or disease progression), the dose modification and monitoring recommendations in the below table should be followed.

Regorafenib is a UGT1A1 inhibitor. Mild, indirect (unconjugated) hyperbilirubinemia may occur in participants with Gilbert's syndrome.

Table 5:

Observed Elevations	1st Occurrence	Restart	Recurrence
AST and/or ALT ≤ 5x ULN (< G3)	Continue dosing, with weekly monitoring of liver function until transaminases return to < 3x ULN (≤ G1) or baseline.		
ALT and/or AST > 5x ULN (≥ G3)	Interrupt dosing, with weekly monitoring until transaminases return to < 3x ULN or baseline.	If the potential benefit for reinitiating regorafenib is considered to outweigh the risk of hepatotoxicity: Reduce 1 dose level and measure serum liver tests weekly for at least 4 weeks.	Discontinue
ALT and/or AST > 20x ULN (≥ G4)	Discontinue		
ALT and/or AST > 3X ULN (≥Grade 2) with concurrent bilirubin > 2 X ULN	Discontinue treatment and measure serum liver tests weekly until resolution. Exception: Patients with Gilbert's syndrome who develop elevated transaminases should be managed as per the recommendations outlined above for ALT/AST elevations.		

NOTE: ALT, AST, and bilirubin must be obtained at baseline and monitored weekly for the first 2 cycles even if values are normal. The investigators may use investigational site or local laboratory AST, ALT, and bilirubin values for subject treatment decisions for day 8, 15 and 22 of regorafenib treatment for Cycles 1 and 2

ALT = alanine aminotransferase, AST = aspartate aminotransferase, NCI-CTCAE v 4.03 = National

Cancer Institute-Common Terminology Criteria for Adverse Events version 4.03, ULN = upper limit of normal.

In **Arm B**, for all other events related to either imatinib or regorafenib (excluding skin toxicity, treatment emergent hypertension, and hepatic dysfunction), see table below:

(Table on next page).

Table 6:

Toxicity	Grade 1	Grade 2	Grade 3	Grade 4
Non-haematologic	Continue at the same dose level	Continue at the same dose level	Withhold dose until toxicity is grade ≤1, then resume at same dose level. If event recurs, withhold dose until toxicity is grade ≤ 1 and reduce dose for next cycle by 1 level. Dose reduce by 1 further level for further grade 3 or higher toxicity.	Withhold dose until toxicity is grade ≤1, then reduce dose for next cycle by 1 level. Dose reduce by 1 further level for further grade 3 or higher toxicity.
Haematologic (excluding anaemia)	Continue at the same dose level.	Continue at the same dose level	Withhold dose until neutrophils ≥1.0 and platelets ≥100, then resume treatment at the same dose level. If event recurs, withhold dose until neutrophils ≥1.0 and platelets ≥100, and reduce dose for next cycle by 1 level. Dose reduce by 1 further level for further grade 3 or higher toxicity.	Withhold dose until neutrophils ≥1.0 and platelets ≥100, then reduce dose for next cycle by 1 level. Dose reduce by 1 further level for further grade 3 or higher toxicity.

7.3. Concomitant Medications/Treatments

All therapies which are considered necessary for the participant's welfare, and which are not expected to interfere with the evaluation of the study drug, may be given at the discretion of the treating clinician.

7.3.1. Recommended

The following medications and treatments are <u>recommended</u> in this study for the prevention and treatment of skin rash including hand-foot skin reaction in those on the alternating arm (Arm B):

Before initiating treatment:

- Check condition of hands and feet

During treatment:

- Avoid pressure points and protect tender areas by use of cushion inserts and well-padded footwear
- Avoid items that rub, pinch, or create friction

- Foot soaks with tepid water and Epsom salts
- Suggest a manicure/pedicure, when indicated
- Recommend pumice stone use for callus or 'rough spot' removal
- Use socks/gloves to cover moisturising creams once applied

Use of creams:

- Apply non-urea based skin-hydrating creams liberally.
- Keratolytic creams: Use sparingly and only to affected (hyperkeratotic) areas.
- Urea-based creams, Salicylic acid 6%, Alpha hydroxy acid (AHA) based creams: Concentrations of approximately 5-8% provide gentle chemical exfoliation. Apply liberally two times each day.
- Topical analgesics like lidocaine 2% should be considered for pain control.
- Topical corticosteroids should be considered for participants with grade 2 or 3 hand-foot skin reaction.

7.3.2. Permitted

The following medications and treatments are permitted in this study:

- Corticosteroids
- Other palliative treatments, such as non-emergency surgery provided the circumstances of which have previously been discussed with the Study Chair (or Delegate).
- Other concomitant therapies considered necessary for the participant's well being may be prescribed at the Investigator's discretion including antiemetics, antidiarrhoeals, anti-inflammatory agents, and analgesics.
- Treatment with non-conventional therapies (for example herbs or acupuncture) and vitamin/mineral supplements is acceptable provided that they do not interfere with the study endpoints in the opinion of the Investigator.
- Bisphosphonates
- Prophylactic or therapeutic treatment with heparin as required.

7.3.3. Use with caution

Pharmacokinetic data from a clinical probe substrate study indicated that regorafenib may be given concomitantly with substrates of CYP2C19 (e.g. omeprazole), CYP2C8 (e.g. rosiglitazone), CYP2C9 (e.g. S-warfarin) without a clinically meaningful drug interaction. Specific caution should be employed when considering or administering a concomitant medication that is metabolized by the phase II glucuronosyl transferases UGT1A1 and 1A9. Studies have shown regorafenib may increase systemic exposure to UGT1A1 and 1A9 substrates.

Co-administration of regorafenib may increase the plasma concentrations of BCRP substrates (e.g., methotrexate, rosuvastatin, fluvastatin, atorvastatin). Therefore, it is recommended to monitor patients closely for signs and symptoms of increased exposure to BCRP substrates

A list of CYP substrates, inhibitors, and inducers is provided at the following website: http://medicine.iupui.edu/clinpharm/ddis/.

- Participants taking narrow therapeutic index medications (e.g.: quinidine, cyclosporine,) should be monitored proactively.
- Co-administration of regorafenib with digoxin has no effect on plasma digoxin.

7.3.4. Prohibited

The following medications should **not** be used during this study:

- Systemic anticancer therapy including cytotoxic therapy, signal transduction inhibitors (including tyrosine kinase inhibitors), immunotherapy, hormonal therapy for cancer, and experimental or unapproved therapies
- Bone marrow transplant or stem cell rescue
- Use of biological response modifiers, such as granulocyte colony stimulating factor (G-CSF), within 3 weeks prior to randomisation. Note: G-CSF may be used during the study in the management of acute toxicity such as febrile neutropenia when clinically indicated or at the discretion of the Investigator; however, they may not be substituted for a required dose reduction. Routine use of G-CSF is not encouraged.
- Radiotherapy
- All traditional/alternative medicines with an anti-cancer indication, including Traditional Chinese Medicine (TCM).
- Strong cytochrome P (CYP) CYP3A4 inhibitors (eg clarithromycin, indinavir, itraconazole, ketoconazole, nefazodone, nelfinavir, posaconazole, ritonavir, saquinovir, telithromycin, voriconazole) or strong CYP3A4 inducers (eg carbamazepine, phenobarbitol, phenytoin, rifampicin, St John's wort). Co-administration of strong CYP3A4 inhibitors may lead to increased toxicity of regorafenib.
- Grapefruit juice

Subjects who require treatment with any of these agents will need to discontinue study treatment, this should be discussed with the Study Chair prior to use.

7.3.5. Concomitant medication reporting

Concomitant medications will not be recorded during the study, except for medications being taken at the onset of serious adverse events or medications known to interact with the study medications.

7.4. Treatment discontinuation

Study treatment will be permanently discontinued for any of the following reasons:

- Progressive disease (PD) is documented by a site investigator.
- Unacceptable toxicity as determined by the patient or site investigator or as defined in section 7.2.
- Delay of day 1 treatment for >28 days due to treatment-related adverse events. For delays >28 days due to reasons other than treatment-related adverse events, please contact the regional co-ordinating centre to discuss treatment continuation.
- The investigator determines that continuation of treatment is not in the patient's best interest.
- Required use of a concomitant treatment that is not permitted, as defined in section 7.3.4.
- Failure to comply with the protocol. If a patient has failed to attend scheduled assessments in the study, the clinician must attempt to determine the reason(s).
- The patient declines further study treatment.

The reasons for discontinuing treatment will be documented in the subject's medical record and eCRF.

Follow up of subjects who stop study treatment should continue according to this protocol (see section 8.1).

7.5. Subsequent treatment

Treatment after discontinuation of study treatment is at the discretion of the patient's clinician.

©NHMRC Clinical Trials Centre

8. ASSESSMENT PLAN

8.1. Schedule of assessments

Assessments must be completed within 3 days of the assessment due date (unless otherwise specified in the footnotes)

ARM A IMATINIB ALONE NB: 1 cycle in continuous treatment arm = 8 weeks of imatinib (56 days)

Parameter	Screening/ Baseline (28 days prior to randomisation)	Cycle 1		Cycle 2		Cycle 3		Cycle 4 and ongoing cycles	30 day safety assessment	Follow-up (12 weekly)
		Wk 1D1	Wk 4 D1	Wk 1D1	Wk 4 D1	Wk1 D1	Wk 4 D1	Wk1 D1		
Informed consent	x[a]									
Pregnancy test	x	x[d]								
Clinical assessment[b]	x	x	x	x	x	x	x	x		
Mutation status if not previously determined	x[c]									
Biocheme[e]	x	x[d]	x	x	x	x	x	x		
Haem[f]	x	x[d]	x	x	x	x	x	x		
Dipstick urine[g]	x	x		x		x		x		
ECG	x									
Imatinib plasma level[h]			x		x					
Blood for translational research[i]	x	x	x		x			x<br>C4 Wk1 D1 only<br>C8 Wk1 D1 only		x<br>At relapse
Adverse event assessment		x	x	x	x	x	x	x	x	
CT scan[j]	x (21 days)			x		x		x		x<br>Continued as per the protocol until PD

a: up to 90 days prior to randomisation

b: clinical assessment including vital signs, blood pressure, physical examination, ECOG PS,

c: analysis of tumour mutation status if not previously known does not need to be completed prior to randomisation

d: if screening bloods and urine tests are performed within 14 days prior to randomisation baseline bloods and urine are not required to be repeated

e: biochemistry including serum creatinine, urea, LFT, TFT, calcium, magnesium, phosphate. INR should be checked at baseline and then as clinically indicated

f: full blood count including differential cell count

g: dipstick urinalysis using standard urine test strip is required on D1 of each cycle. If abnormal, further investigations should be conducted as clinically appropriate

h: blood collected for drug (imatinib) level testing. Refer to Biological Sampling Handbook for details of collection and processing. Documentation of compliance with treatment during the preceding cycle should be documented in the eCRF

i: blood for **optional** translational research will be collected at these timepoints. Refer to Biological Sampling Handbook for details of collection and processing

j: for the first 12months, CT scans are performed 8 weekly from randomisation after which CT scans are performed 12 weekly. Imaging of the brain should be undertaken if clinically indicated. CT scans should continue as per the protocol until PD

 ©NHMRC Clinical Trials Centre

ARM B IMATINIB ALTERNATING WITH REGORAFENIB

NB: 1 cycle in alternating arm = 21-25 days of imatinib, 3-7day washout, 21 days of regorafenib, 7 day washout

Parameter	Screening/ Baseline (28 days prior to randomisation)	Randomis-ation	Cycle 1				Cycle 2				Cycle 3	Cycle 4 and ongoing cycles	30 day safety assessment	Follow-up (12 weekly)
			Wk1 D1	Last day of Imatinib	Wk5 D1	Last day of Regor	Wk1 D1	Last day of Imatinib	Wk5 D1	Last day of Regor	Wk1 D1 & Wk5 D1	Wk1 D1		
Informed consent	x[a]													
Pregnancy test	x	If patient is on Imatinib therapy for longer than 21 days prior to randomisation on a washout must occur. Imatinib must stop for a minimum of 3 days prior to starting regorafenib on D29 .	x[d]											
Clinical assessment[b]	x		x		x		x		x		x	x		
Additional monitoring -BP					x[m]		x[m]		x[m]					
Mutation status if not previously determined	x[c]													
Biocheme[e]	x		x[d]		x		x		x		x	x		
AST, ALT and bilirubin[h]					x[h]	x[h]			x[h]	x[h]				
Haem[f]	x		x[d]		x		x		x		x	x		
Dipstick urine[g]	x		x				x				x Wk1 D1 only	x		
ECG	x													
Imatinib plasma level [i]				x				x						
Regorafenib plasma level [j]						x				x				
Blood for translational research[k]	x		x	x		x		x		x		x C4 Wk1 D1 & C8 Wk1 D1 only		x At relapse
Adverse event assessment			x		x		x		x		x	x	x	
CT scan[l]	x (21 days)						x				x Wk1 D1 only	x		x Until PD
PET Scan[n] -Imatinib washout								x	x					
or PET Scan [n] -Regorafenib washout										x	x Wk1 D1 only			

a: up to 90 days prior to randomisation
b: clinical assessment including vital signs, blood pressure, physical examination, ECOG PS
c: analysis of tumour mutation status if not previously known does not need to be completed prior to randomisation
d: if screening bloods and urine test are performed within 14 days prior to randomisation baseline bloods and urine tests are not required to be repeated
e: biochemistry including serum creatinine, urea, LFT, TFT, calcium, magnesium, phosphate. INR should be checked at baseline and then as clinically indicated
f: full blood count including differential cell count
g: dipstick urinalysis using standard urine test strip is required on D1 of each cycle. If abnormal, further investigations should be conducted as clinically appropriate

©NHMRC Clinical Trials Centre

h: Liver function testing must also be performed weekly in the alternating treatment arm during regorafenib use for the first 2 cycles. AST, ALT and bilirubin must be monitored weekly during regorafenib use for the first 2 cycles of the patient taking regorafenib. Site or local laboratory may be used.
i: blood collected for drug (imatinib) level testing. Refer to Biological Sampling Handbook for details of collection and processing. Documentation of compliance with treatment during the preceding cycle should be documented in the eCRF
j: blood collected for drug (regorafenib) level testing. Refer to Biological Sampling Handbook for details of collection and processing. Documentation of compliance with treatment during the preceding cycle should be documented in the eCRF
k: blood for **optional** translational research will be collected at these timepoints. Refer to Biological Sampling Handbook for details of collection, processing and storage
l: for the first 12months, CT scans are performed 8 weekly from randomisation after which CT scans are performed 12 weekly. Imaging of the brain should be undertaken if clinically indicated. CT scans should continue as per the protocol until PD
m: weekly blood pressure measurements are required from the commencement of regorafenib in cycle 1 until the completion of cycle 2.
n – only in those Arm B patients enrolled in the PET substudy. Patients randomised to have two scans and timing is based on a second randomisation - either PET scans during imatinib washout period, or regorafenib washout period. Depending on allocated arm, PET scans to be performed at the completion of regorafenib or imatinib tablets (or up to 2 days prior) and before the commencement of the subsequent imatinib or regorafenib cycle (or up to 2 days prior). These scans are to be performed in Cycle 2 at the earliest or in a subsequent cycle (at the first opportunity after a complete cycle of treatment with no dose modifications is given).

©NHMRC Clinical Trials Centre

8.2. Assessment phase definitions and special circumstances

8.2.1. Baseline

Some patients in this study may have already commenced treatment with imatinib, while others will be treatment naive.

- Informed consent within 90 days
- Medical History, previous anti-cancer therapies and physical assessment
- ECG
- Blood collection for biochemistry including serum creatinine, urea, LFT, , calcium, magnesium, phosphate, TFT, full blood count including differential cell count and INR
- Note: If screening bloods are performed within 14 days of randomisation baseline bloods are not required.
- Urinalysis
- Pregnancy test
- ECG
- CT scan chest / abdominal / pelvis within 21 days of randomisation.
- Optional blood for research

8.2.2. During treatment

- Clinical assessment (including vital signs, blood pressure, physical examination, ECOG PS, adverse event monitoring), full blood count (including differential cell count), biochemistry (including serum creatinine, urea, LFT, calcium, magnesium, phosphate) to be performed for ARM A at the beginning of each cycle and at C1, C2 and C3 Wk 4 D1, for ARM B at the beginning of each cycle and at C1, C2 and C3 Wk 5 D1
- All participants who are on regorafenib should have their blood pressure monitored weekly from the commencement of regorafenib in cycle 1 until the end of cycle 2. During this time, participants must attend the clinic weekly for their blood pressure to be taken. Alternatively, the weekly blood pressure measurements can be done by GPs, or using home blood pressure monitors if available. Blood pressure readings collected by the patient at home or by their GP will need to be communicated to the study site for appropriate management. Recommendations for anti-hypertension management will be provided in the Study Manual.
- Liver function testing must also be performed weekly in the alternating treatment arm (Arm B) during regorafenib use for the first 2 cycles. INR should be checked at baseline and then as clinically indicated.
- Dipstick urinalysis using standard urine test strip is required on day 1 of each cycle. If abnormal, further investigations should be conducted as clinically appropriate.
- For patients on Arm A blood collection for drug (imatinib) level testing to be performed on cycle 1 week 4 and cycle 2 week 4. For patients on the alternating regimen (Arm B) blood collected for drug (imatinib) level testing to be performed on cycle 1, on last day of imatinib administration and cycle 2 on last day of imatinib administration. Blood collected for drug (regorafenib) level testing to be performed on cycle 1 week 7 (on last day of regorafenib) and cycle 2 week 7 (on last day of regorafenib).
- Blood for translational research will be collected at the following timepoints;
    - Baseline/screening
    - C1 Wk1 D1 (prior to the commencement of study treatment, Baseline)
    - For Arm A: C1 Wk4 and C2 Wk4.

  - For Arm B: C1 on the last day of imatinib administration C1 Wk7 (on the last day of regorafenib administration)
  - For Arm B: C2 on the last day of imatinib administration and C2 Wk7 (on the last day of regorafenib administration)
  - C4, and 8 Wk1 D1
  - Upon relapse
- For the first 12months, CT scans are performed 8 weekly from randomisation after which CT scans are performed 12 weekly until progression.
- A select group of Arm B patients will be enrolled in the PET substudy – Arm B patients will be further randomised to have PET scans either during the imatinib washout period, or during the regorafenib washout period. PET will be carried out on 2 occasions in each group and will be performed in Cycle 2 at the earliest, or in a subsequent cycle (at the first opportunity after a complete cycle of treatment without dose modifications is given).

8.2.3. End of treatment

No additional investigations are required for this trial at the time of treatment cessation.

8.2.4. 30 day safety assessment

A safety assessment should be performed to include any adverse events occurring within 30 days (+/- 7 days) after the last dose of study treatment.

8.2.5. Follow-up after treatment

Subjects who stop study treatment prior to the time recommended in the protocol will continue follow-up visits.

If a patient wishes to stop the study visits, they will be requested to allow their ongoing health status to be periodically reviewed via continued study visits or phone contact or from their general practitioner, or medical records, state-based cancer registries and/or the national mortality registry.

8.2.6. After study is closed

The study will be closed after data required has been collected and analysed. Collection of long term outcomes, for example survival data, may continue after the main study is closed, using a simple follow-up CRF, monitoring of central registries, or contact with treating clinicians.

9. OUTCOMES, ENDPOINTS AND OTHER MEASURES

9.1. Objective tumour response (complete or partial response) at or before 9 months

Tumour evaluation using the RECIST Version 1.1 guidelines will be used to determine response status (i.e. complete response, partial response, stable disease, and progressive disease) at each assessment time point. The OTRR will be calculated by summing the number of participants assessed as having a complete or partial response within the first 9 months from the time from either (i) randomization (if patients have not yet commenced treatment) or (ii) commencement of therapy (if patients are randomized during the first cycle of imatinib)., and dividing this by the total number of participants evaluable for response. For patients who undergo surgery, the best response is determined in the time period that precedes the date of surgery

9.2. *Progression free survival* *(disease progression or death)*

The length of time from from either (i) randomization (if patients have not yet commenced treatment) or (ii) commencement of therapy (if patients are randomized during the first cycle of imatinib)until disease progression. Disease progression is defined according to RECIST 1.1

9.3.Clinical benefit rate at 3 cycles (24 weeks)

The clinical benefit rate will be calculated by summing the number of participants assessed as having a complete response, partial response or stable disease within the first 24 weeks from randomisation, and dividing this by the total number of participants evaluable for response (according to RECIST Version 1.1) within the first 24 weeks.

9.4. Time to treatment failure

Time to treatment failure is defined as the time from either (i) randomization (if patients have not yet commenced treatment) or (ii) commencement of therapy (if patients are randomized during the first cycle of imatinib) to treatment discontinuation for any reason, including disease progression, treatment toxicity, patient preference, or death.

9.5. Adverse Events (worst grade according to NCI CTCAE v4.03)

The NCI Common Terminology Criteria for Adverse Events version 4 (NCI CTCAE v4.03) will be used to classify and grade the intensity of adverse events after each treatment cycle.

9.6. Overall survival

Overall survival is defined as the interval from either (i) randomization (if patients have not yet commenced treatment) or (ii) commencement of therapy (if patients are randomized during the first cycle of imatinib) to date of death from any cause, or the date of last known follow-up alive.

9.7. Rate of patients having macroscopically complete removal of all residual disease by surgery

This is defined as the rate of patients who proceed to surgery with the aim of resecting all remaining macroscopic disease.

9.8. Change in PET imaging during washout period of regorafenib and imatinib in those taking part in the PET substudy

The PET substudy aims to primarily evaluate the change in PET response during washout periods for regorafenib and imatinib. PET scans will be performed in a subgroup of participants enrolled to alternating treatment (Arm B) at selected centres. These patients will be further randomised to have PET scans either during the imatinib washout period, or during the regorafenib washout period. At these selected centres, PET scans will be conducted in addition to regular imaging studies. In the sub-study, PET will be carried out on 2 occasions in each group in Cycle 2 at the earliest, or in a subsequent cycle (at the first opportunity after a complete cycle of treatment without dose modification is given). For those randomised to PET scans during the imatinib washout period, a PET scan will be performed immediately following the last dose of imatinib (or up to 2 days prior) and before the first regorafenib dose (or up to 2 days prior). For those randomised to PET scans during the regorafenib washout period, a PET scan will be performed immediately following the last regorafenib dose (or up to 2 days prior) and on the first day of the subsequent imatinib cycle prior to the first dose of imatinib (or up to 2 days prior). The change between the PET standardised uptake values (SUV) of the scan performed at

the commencement of the drug washout period and the PET SUV of the scan performed at the conclusion of the drug washout period will be assessed.

9.9. Biomarkers

This will include investigations of how regorafenib and imatinib may work in people with GIST, biological mechanisms of action as well as studies that may help to understand the pathogenic course of this cancer and related diseases.

Planned tissue biomarker analyses may include but are not limited to: *KIT*/PDGFRA mutation analysis and other proteins relating to PDGFR signalling.

Planned analysis of blood collected for research may include but not be limited to:

- circulating serum/plasma growth factor and cytokine levels (multiplex assay)
- imatinib plasma levels and in Arm B regorafenib plasma
- circulating tumour cells and
- KIT/PDGFRA mutations in circulating blood DNA
- circulating DNA load

Since the identification of new biomarkers correlating with disease activity and the efficacy or safety of treatment are rapidly evolving, the definitive list of biomarkers remains to be determined.

10. SAFETY REPORTING

10.1. Definitions

An ADVERSE EVENT (AE) is any untoward medical occurrence in a patient or clinical investigational subject administered a pharmaceutical product and which does not necessarily have a causal relationship with this treatment. An AE can therefore be any unfavourable or unintended sign (including an abnormal laboratory finding), symptom, or disease temporally associated with the use of a medicinal investigational product, whether or not considered related to the medicinal product (see below).

Adverse events include the following:

- All suspected adverse drug or device reactions
- All reactions from drug or device – overdose, abuse, withdrawal, sensitivity, toxicity or failure of expected pharmacological action (if appropriate)
- Apparently unrelated illnesses, including the worsening (severity, frequency) of pre-existing illnesses
- Injury or accidents.
- Abnormalities in physiological testing or physical examination that require clinical intervention or further investigation (beyond ordering a repeat examination)
- Laboratory abnormalities that require clinical intervention or further investigation (beyond ordering a laboratory test).

Any untoward event that occurs after the protocol-specified reporting period which the Investigator believes may be related to the drug or device.

A SERIOUS ADVERSE EVENT (SAE) is any untoward medical occurrence that at any dose:

- results in death,

- is life-threatening (i.e. the subject is at risk of death at the time of the event),
- requires inpatient hospitalisation or prolongation of existing hospitalisation,
- results in persistent or significant disability or incapacity,
- is a congenital anomaly/birth defect,
- other important medical events which, in the opinion of the investigator, are likely to become serious if untreated, or as defined in the protocol

NOTES:

(i) The term "life-threatening" in the definition of "serious" refers to an event in which the patient was at risk of death at the time of the event; it does not refer to an event which hypothetically might have caused death if it were more severe.

(ii) Important medical events which may not be immediately life-threatening or result in death or hospitalization but which may jeopardize the patient or may require intervention to prevent one of the listed outcomes in the definition above should also be considered serious.

A SUSPECTED UNEXPECTED SERIOUS ADVERSE REACTION (SUSAR) is an SAE that is related to the drug or device and is unexpected (i.e. not listed in the investigator brochure or approved Product Information; or is not listed at the specificity or severity that has been observed; or is not consistent with the risk information described in the Subject Information Sheet and Informed Consent Form or elsewhere in the protocol. (FDA, Safety Reporting Requirements for INDs and BA/BE Studies, draft guidance, September 2010)).

An event is causally related if there is a reasonable possibility that the drugs, imatinib or regorafenib caused the AEs, i.e. there is evidence to suggest a causal relationship between the drug and the event.

For the purposes of this study, the following adverse events are not reported as SAEs:

- Hospitalisations related to disease progression
- Hospitalisations for management of pain related to disease
- Any elective surgery not related to cancer treatment
- Surgery for complete resection of residual disease
- Deaths related to disease progression
- Elective hospitalisation to simplify study procedures

10.2. Reporting of Serious Adverse Events (including SUSARs)

The investigator is responsible for reporting all Serious Adverse Events (including SUSARs) occurring during the study to the NHMRC Clinical Trial Centre (CTC) within 1 working day of the investigator becoming aware of the event using the SAE form. All SAEs must be reported from the first dose of study treatment up to 30 days from the end of study intervention.

SAE reports should be reported to the CTC as per the procedure documented in the Study Manual. The investigator must notify their local HREC as required.

The CTC will provide SUSAR reports and SAE line listings to Investigators for submission to Human Research Ethics Committees (HRECs) as required.

For AGITG sites, the CTC will be responsible for providing reports to the Lead HREC as required.

 ©NHMRC Clinical Trials Centre

The local study sponsor in each country/region will submit 'reportable safety events' to the local regulatory authorities (e.g. TGA in Australia, Medsafe in NZ etc.).

The following information will be recorded for each Serious Adverse Event:

- Event description including classification according to NCI CTCAE 4.03
- Severity / Worst Grade
- Attribution to study intervention
- Expectedness (listed in IB/product information),
- Action taken with study intervention
- Type of SAE (e.g. hospitalisation)
- Outcome of SAE including end date if recovered

10.3. Pregnancy

In the event of a pregnancy occurring during the course of the study, the subject must be withdrawn from study drug immediately. Pregnancies occurring up to 6 months after the completion of the study drug must also be reported to the investigator. The investigator should counsel the patient; discuss the risks of continuing with the pregnancy and the possible effects on the foetus.

The local co-ordinating centre must be notified within 1 working day using the SAE form and the subject followed during the entire course of the pregnancy and postpartum period. Parental and neonatal outcomes must be recorded even if they are completely normal.

Pregnancy occurring in the partner of a patient participating in the study and up to 90 days after the completion of the test drug should also be reported to the investigator and the regional co-ordinating centre. The partner should be counselled and followed as described above.

11. CENTRAL REVIEW AND BIOSPECIMEN COLLECTION

11.1. Central Biospecimen Collection

Formalin-fixed paraffin–embedded (FFPE) diagnostic tumour tissue is required from all participants and will be collected for:

- pathology quality control and
- translational research (including potential future translational research relevant to this study).

The tissue will be from archival tumour material – no additional biopsy of the participant is required. Representative FFPE tissue will be collected by sites and sent to a central laboratory for analysis.
Refer to the Biological Sampling Handbook for the details of collection, processing and shipping procedures.

Blood for research will be collected from consenting participants at multiple time points and will be processed for blood components including, but not limited to serum and plasma. Processed samples will then be sent to a central laboratory for analysis. Refer to the Biological Sampling Handbook for the details of collection, processing and shipping procedures.

11.2. Central Imaging Collection

Central review of CT reports will occur retrospectively and will be used for final endpoint classification for the study. A subset of CT scans may be collected on CD/DVD so these can be reviewed centrally.

Refer to the Study Manual for collection requirements

12. TREATMENT INFORMATION

12.1. Description of Study Products

The regorafenib 40 mg tablet is coated, not divisible, grey-orange-red, oval (length 16 mm, width 7 mm, thickness 4.9-5.6 mm) and 472 mg each in total weight. The packaging configuration is 30 tablets and a 3g desiccant capsule per bottle of regorafenib 40 mg.

Tablets are in an immediate-release dosage form with rapid dissolution characteristics under in vitro test conditions.

The regorafenib 40 mg tablet contains regorafenib and the inactive excipients microcrystalline cellulose, croscarmellose sodium, magnesium stearate, povidone, colloidal anhydrous silica, polyvinyl alcohol-part hydrolyzed, talc, titanium dioxide E171 (color index 77891), macrogol/PEG 3350, lecithin (soy), iron oxide yellow E172 (color index 77491), and iron oxide red E172.

Regorafenib tablets will be packaged separately in high-density polyethylene (HDPE) bottles with a white child-resistant closure and induction seal. Each bottle includes a desiccant. The bottles will have a label affixed containing study identification, quantity of tablets, and other details as required by local regulations. Once the drug has been received, it must be kept in a secure, dry location at a temperature not above 25 °C (77 °F).

Imatinib is manufactured as 100mg and 400mg tablets packaged in polyethylene bottles. Once the drug has been received, it must be kept in a secure, dry location at a temperature not above 30 °C.

The tablets contain imatinib, and the excipients microcrystalline cellulose, crospovidone, hypromellose, anhydrous colloidal silica, magnesium stearate, iron oxide yellow CI 77492, iron oxide red CI 77491, macrogol 4000, and talc.

12.2. Supply of Study Product

Bayer will be providing regorafenib for the study and the regional co-ordinating centre will arrange for the delivery of the investigational product to sites. Imatinib will be supplied according to usual hospital practice.

12.3. Drug Accountability

The Pharmacy Department at participating institutions will maintain a record of drugs dispensed for each patient and subsequent returns. The Pharmacy will also maintain a record of drug receipt and drug destruction as appropriate.

13. STATISTICAL CONSIDERATIONS

13.1. Sample Size

This is a non-comparative randomized phase II design to investigate the efficacy/activity of an alternating regimen of 21-25 days of imatinib followed by a 3-7-day gap for washout followed by 21 of regorafenib and a 7-day gap for washout. The control group will be a regimen of continuous imatinib in a 56 day cycle. In order to demonstrate clinical activity, the response rate at 9 months is deemed to be an appropriate endpoint. Study and resource constraints limit the sample size in the alternating regimen to 38 patients and as such the measured activity will be primarily descriptive. The anticipated sample size of the study will be 60 evaluable patients in an unequal randomisation ratio

average approximately 2:1 randomisation (22 in the control and 38 in the alternating group). Based on a 95% one-sided confidence interval (Mehta and Cain, *JCO* 1984; 2 (6):676-681), for different levels of the true underlying response rate, the table below gives the minimum number of responses required to be observed in the alternating group. Accrual may continue beyond 60 patients, but any increase would be modest.

	True response rate (%)			
	30	40	50	60
Number of responses	≥ 7	≥ 10	≥ 13	≥ 17

13.2. Statistical Analysis

Analyses will include all patients who were randomised. Treatment activity will be assessed by the proportion, together with the 95% confidence interval, of patients having an objective response at or before 9 months. Similar proportions will be determined in the control group,

Time-to event endpoints of progression free and overall survival and time to treatment failure will be described using the method of Kaplan-Meier. Treatment failure is defined as the first event before 12 months of death, disease progression and unacceptable toxicity causing study treatment to cease or patient preference to cease therapy. For purposes of treatment failure, only events in the first 12 months will be considered. Patients not experiencing a treatment failure within 12 months will be censored at 12 months. While there will not be any formal comparisons between the two groups, exploratory comparative analyses may be performed. For time-to-event outcomes, these will include univariate logrank and multivariate proportional hazards comparisons. Other exploratory analyses will also be performed as appropriate.

13.3. Compliance Assessment

After 15 participants are enrolled in the experimental arm and have received at least 2 cycles of treatment, an interim analysis examining the dose intensity of imatinib and regorafenib will be performed. Modifications to the protocol will be considered if more than 10 patients have not received more than 75% of the planned cumulative doses in the experimental arm. This would be inconsistent with a 50% compliance rate. While the interim analysis is being performed, the study will continue recruiting.

14. STUDY ORGANISATION

This trial is conducted as an intergroup collaboration between the Australasian Gastrointestinal Trials Group (AGITG), the Scandinavian Sarcoma Group (SSG), and the Soft Tissue Sarcoma Group of the European Organisation for Research and Treatment of Cancer (EORTC).
SSG/EORTC will oversee the management of the study in Scandinavia and Europe and AGITG/NHMRC CTC in ANZ and Asia. Interpretation of data and publication will be a joint collaboration.
Randomisation, database development, management and statistical analysis will be conducted at the NHMRC CTC.

14.1. Trial Management Committee(s)

A locally formed Trial Management Committee (TMC) in each region will monitor the progress of the study and review of information from related research, and implementation of recommendations from

other study committees and external bodies (e.g. ethics committees). A member from the TMC will represent each region on the International Steering Committee (ISC).

14.2. Independent Safety and Data Monitoring Committee

The AGITG Independent Data, Safety and Monitoring Committee (IDSMC) will closely monitor the trial for safety and toxicity. Apart from the interim analysis examining dose intensity in 13.3 no formal interim analysis of efficacy is planned but the IDSMC may review outcome data (PFS and OS) if it is felt appropriate. The IDSMC will pay particular attention to the rate of participant drop-out from trial (especially in the experimental arm) for reasons of toxicity or participant/clinician preference.

The ISDMC will provide independent assessment of patient safety and trial progress, making recommendations to the TMC about the continuation of the trial based on data made available by the trial statistician. Additionally, the IDSMC will review all SUSARS in a timely manner after appropriate authorities have been notified as detailed in section 10.

15. ADMINISTRATIVE ASPECTS

For European and Scandinavian sites, please refer to the EORTC/SSG Group Specific Appendix (GSA) for all practical and administrative aspects of the protocol specific to the SSG/EORTC (organization, specific scientific information, authorization procedure, registration/randomization, quality, ethics, responsibilities, sponsorship and financing, insurance and publication policy).

15.1. Ethics and regulatory compliance

This study will be conducted according to the Note for Guidance on Good Clinical Practice (CPMP/ICH/135/95) annotated with TGA comments (Therapeutic Goods Administration DSEB July 2000) and in compliance with applicable laws and regulations. The study will be performed in accordance with the NHMRC Statement on Ethical Conduct in Research Involving Humans (© Commonwealth of Australia 2007), and the NHMRC Australian Code for the Responsible Conduct of Research (©Australian Government 2007), and the principles laid down by the World Medical Assembly in the Declaration of Helsinki 2008. To this end, no patient will be recruited to the study until all the necessary approvals have been obtained and the patient has provided written informed consent. Further, the investigator shall comply with the protocol, except when a protocol deviation is required to eliminate immediate hazard to a subject. In this circumstance the NHMRC CTC, principal investigator and HREC must be advised immediately.

15.2. Confidentiality

The study will be conducted in accordance with applicable Privacy Acts and Regulations. All data generated in this study will remain confidential. All information will be stored securely at the relevant coordinating centre and will only be available to people directly involved with the study and who have signed a Confidentiality Agreement. No personal information that reveals the patient identity will be entered into the CRFs or the study database. The patients will be provided a study code (subject number) that links the patient identity to the information stored in the study database.

15.3. Protocol amendments

Changes and amendments to the protocol can only be made by the International Steering Committee. Approval of amendments by the Institutional HREC is required prior to their implementation. In some instances, an amendment may require a change to a consent form. The Investigator must receive

approval/advice of the revised consent form prior to implementation of the change. In addition, changes to the data collected, if required, will be incorporated in the amendment.

The investigator should not implement any changes to, or deviations from, the protocol except where necessary to eliminate immediate hazard(s) to trial subject(s).

15.4. Data Handling and Record Keeping

All trial data required for the monitoring and analysis of the study will be recorded on the CRFs provided. All required data entry fields must be completed. Data corrections will be done according to the instructions provided. The investigator will be asked to confirm the accuracy of completed CRFs by signing key CRFs as indicated.

Source documents pertaining to the trial must be maintained by investigational sites. Source documents may include a subject's medical records, hospital charts, clinic charts, the investigator's subject study files, as well as the results of diagnostic tests such as X-rays, laboratory tests, and electrocardiograms. The investigator's copy of the case report forms serves as part of the investigator's record of a subject's study-related data.

The following information should be entered into the subject's medical record:

a. Subject’s name, contact information and protocol identification.

b. The date that the subject entered the study.

c. A statement that informed consent was obtained (including the date).

d. Relevant medical history

e. Dates of all subject visits and results of key trial parameters.

f. Occurrence and status of any adverse events.

g. The date the subject exited the study, and a notation as to whether the subject completed the study or reason for discontinuation.

All study-related documentation at ANZ sites will be maintained for 15 years following completion of the study.

15.5. Audit and Inspection

This study may be subject to audit or inspection by representatives of the collaborative group or the CTC/SSG/EORTC or Bayer Healthcare Pharmaceutical or representatives of regulatory bodies (e.g. Therapeutic Goods Administration (TGA).

15.6. Clinical Study Report

A Clinical Study Report which summarises and interprets all the pertinent study data collected will be issued which may form the basis of a manuscript intended for publication.

The ISC will appoint a Writing Committee to draft manuscript(s) based on the trial data. Manuscript(s) will be submitted to peer-reviewed journal(s). The first publication will be the report of the full trial results based on the main protocol in the Group name with subsequent publications of data subsets in individual names based on contribution. The Writing Committee will develop a publication plan, including authorship, target journals and expected dates of publication. All publications (including substudies) must receive prior written approval from the ISC prior to submission.

16. REFERENCES

1. Verweij J, Casali P, Zalcberg J et al. Progression-free survival in gastrointestinal stromal tumours with high dose imatinib: randomised trial. Lancet. 25 September 2004; 364 (9440):1127 – 1134
2. Blanke CD, Rankin C, Demetri GD et al. Phase III randomized, intergroup trial assessing imatinib mesylate at two dose levels in patients with unresectable or metastatic gastrointestinal stromal tumors expressing the kit receptor tyrosine kinase: S0033. J Clin Oncol. Feb 1 2008; 26(4):626-32
3. von Mehren M.Follow-up results after 9 years (yrs) of the ongoing, phase II B2222 trial of imatinib mesylate (IM) in patients (pts) with metastatic or unresectable KIT+ gastrointestinal stromal tumors (GIST) (abstract). J Clin Oncol 2011; 29(15_Suppl): Abstract 10016.
4. Sleijfer S, Wiemer E, Seynaeve C et al. Improved insight into resistance mechanisms to imatinib in gastrointestinal stromal tumors: a basis for novel approaches and individualization of treatment. Oncologist. June 2007; 12(6):719-26.
5. Antonescu CR, Besmer P, GuoT, et al. Acquired resistance to imatinib in gastrointestinal stromal tumor occurs through secondary gene mutation. Clin Cancer Res 2005; 11:4182-90.
6. Desai J, Shankar S, Heinrich MC. Clonal Evolution of Resistance to Imatinib in Patients with Metastatic Gastrointestinal Stromal Tumors**.** Clin Cancer Res. 15 September 2007; 13:5398 - 5405
7. Heinrich MC, Griffith DJ, Druker BJ et al. Inhibition of c-kit receptor tyrosine kinase activity by STI 571, a selective tyrosine kinase inhibitor. Blood. 2000; 96:925-32
8. Demetri GD, von Mehren M, Blanke CD et al. Efficacy and safety of imatinib mesylate in advanced gastrointestinal stromal tumors. N Engl J Med. 2002; 347: 472-80
9. Bardsley MR, Horvath VJ, Asuzu DT, et al. Kit^{low} stem cells cause resistance to Kit/platelet-derived growth factor α inhibitors in murine gastrointestinal stromal tumors. Gastroenterology 2010; 139: 942–52
10. George S, Wang Q, Heinrich M et al. Efficacy and Safety of Regorafenib in Patients With Metastatic and/or Unresectable GI Stromal Tumor After Failure of Imatinib and Sunitinib: A Multicenter Phase II Trial. J Clin Onc. May 21 2012; 30(19): 2401-07
11. Bono P, Krause A, von Mehren M et al. Serum KIT and KIT ligand levels in patients with gastrointestinal stromal tumors treated with imatinib. Blood. 15 April 2004; 103(8):2929-35
12. Wilhelm S, Dumas J, Adnane L et al. Regorafenib (BAY 73-4506): A new oral multikinase inhibitor of angiogenic, stromal and oncogenic receptor tyrosine kinases with potent preclinical antitumor activity. Int J Cancer. 1 July 2011; 129(1):245-55
13. Demetri G, Reichardt P, Kang YK et al. Efficacy and safety of regorafenib for advanced gastrointestinal stromal tumours after failure of imatinib and sunitinib (GRID): an international, multicentre, randomised, placebo-controlled, phase 3 trial. Lancet 2013; 381: 295 -302
14. A'Hern, R. Sample size tables for exact single-stage phase II designs. Stat in Med 2001l 20: 859-866.

17. LIST OF APPENDICES

Appendix 1. National Cancer Institute Common Terminology Criteria for Adverse Events (CTCAE) Version 4.03

Appendix 2. Response Evaluation Criteria in Solid Tumours (RECIST Version 1.1)

©NHMRC Clinical Trials Centre

Appendix 1. National Cancer Institute Common Terminology Criteria for Adverse Events (CTCAE) Version 4.03

Link: http://ctep.cancer.gov/protocolDevelopment/electronic_applications/ctc.htm

©NHMRC Clinical Trials Centre

Appendix 2. Response Evaluation Criteria in Solid Tumours (RECIST Version 1.1)

These instructions are based on the guidelines recommended in Eisenhauer EA, Therasse P, Bogaerts J et al. New response evaluation criteria in solid tumours: Revised RECIST guideline (version 1.1). Eur J Cancer 2009; 45: 228-47.

1 Evaluable for response

All participants who have received at least one cycle of therapy and have their disease re-evaluated will be considered evaluable for response (exceptions will be those who exhibit objective disease progression prior to the end of cycle 1 who will also be considered evaluable). Participants on therapy for at least this period and who meet the other listed criteria will have their response classified according to the definitions set out below.

2 Disease and lesion definitions

2.1 *Measurable Disease*. Measurable *tumour lesions* are defined as those that can be accurately measured in at least one dimension (longest diameter to be recorded) as ≥ 20 mm with chest x-ray, and as ≥10 mm with CT scan or clinical examination. Bone lesions are considered measurable only if assessed by CT scan and have an identifiable soft tissue component that meets these requirements (soft tissue component ≥ 10 mm by CT scan). *Malignant lymph nodes* must be ≥ 15mm in the short axis to be considered measurable; only the short axis will be measured and followed. All tumour measurements must be recorded in millimetres. Previously irradiated lesions are not considered measurable unless progression has been documented in the lesion.

2.2 *Non-measurable Disease*. All other lesions (or sites of disease), including small lesions are considered non-measurable disease. Bone lesions without a measurable soft tissue component, leptomeningeal disease, ascites, pleural/pericardial effusions, lymphangitis cutis/pulmonis, inflammatory breast disease, lymphangitic involvement of lung or skin and abdominal masses followed by clinical examination are all non-measurable. Lesions in previously irradiated areas are non-measurable, unless progression has been demonstrated.

2.3 *Target Lesions*. When more than one measurable tumour lesion is present at baseline all lesions up to *a maximum of 5 lesions in total* (and a maximum of *2 lesions per organ*) representative of all involved organs should be identified as target lesions and will be recorded and measured at baseline. Target lesions should be selected on the basis of their size (lesions with the longest diameter), be representative of all involved organs, but in addition should be those that lend themselves to *reproducible repeated measurements*. Note that pathological lymph nodes must meet the criterion of having a short axis of ≥ 15 mm by CT scan and only the *short* axis of these lymph nodes will contribute to the baseline sum. All other pathological lymph nodes (those with a short axis ≥ 10 mm but <15 mm) should be considered non-target lesions. Nodes that have a short axis < 10 mm are considered non-pathological and should not be recorded or followed (see 10.2.4). At baseline, the sum of the target lesions (longest diameter of tumour lesions plus short axis of target lymph nodes: overall maximum of 5) is to be recorded.

After baseline, a value should be provided on the CRF for all identified target lesions for each assessment, even if very small. If extremely small and faint lesions cannot be accurately measured but are deemed to be present, a default value of 5 mm may be used. If lesions are too small to measure and indeed are believed to be absent, a default value of 0 mm may be used.

2.4 *Non-target Lesions*. All non-measurable lesions (or sites of disease) plus any measurable lesions over and above those listed as target lesions are considered *non-target lesions*. Measurements are not required but these lesions should be noted at baseline and should be followed as “present” or “absent”.

3 Response Definitions

ALT GIST

All participants will have their BEST RESPONSE from the start of study treatment until the end of treatment classified as outlined below:

Complete Response (CR): Disappearance of all *target* and *non-target* lesions and normalisation of any specified tumour markers. Pathological lymph nodes must have short axis measures < 10mm (Note: continue to record the measurement even if < 10mm and considered CR). Residual lesions (other than nodes < 10mm) thought to be non-malignant should be further investigated (by cytology or PET scans) before CR can be accepted. Confirmation of response is sometimes required in studies where objective tumour response is the primary endpoint, and the details of confirmation are then specified in the body of the protocol.

Partial Response (PR): At least a 30% decrease in the sum of measures for target lesions (longest diameter for tumour lesions and short axis measure for target lymph nodes), taking as reference the baseline sum of diameters. Non-target lesions must be non-PD. Confirmation of response is sometimes required in studies where objective tumour response is the primary endpoint, and the details of confirmation are then specified in the body of the protocol.

Stable Disease (SD): Neither sufficient shrinkage to qualify for PR nor sufficient increase to qualify for PD taking as reference the smallest sum of diameters on study.

Progressive Disease (PD): At least a 20% increase in the sum of diameters of measured lesions taking as references the smallest sum of diameters recorded on study (including baseline) AND an absolute increase of ≥ 5mm. Appearance of new lesions will also constitute progressive disease (including lesions in previously unassessed areas). In exceptional circumstances, unequivocal progression of non-target disease may be accepted as evidence of disease progression, where the overall tumour burden has increased sufficiently to merit discontinuation of treatment or where the tumour burden appears to have increased by at least 73% in volume. Modest increases in the size of one or more non-target lesions are NOT considered unequivocal progression. If the evidence of PD is equivocal (target or non-target), treatment may continue until the next assessment, but if confirmed, the earlier date must be used.

Table 1: Integration of Target, Non-target and New lesions into response assessment

**Target Lesions**	**Non-Target Lesions**	**New Lesions**	**Overall Response***	**Best Response for this category also requires**
Target lesions ± non target lesions				
CR	CR	No	CR	Normalisation of specified tumour markers, AND lymph nodes <10mm
CR	Non-CR/Non-PD	No	PR	
CR	Not all evaluated	No	PR	
PR	Non-PD/<br>not all evaluated	No	PR	
SD	Non-PD/<br>not all evaluated	No	SD	Documented at least once ≥ 4 wks. from baseline
Not all evaluated	Non-PD	No	NE	
PD	Any	Any	PD	
Any	PD	Any	PD	
Any	Any	Yes	PD	
Non target lesions ONLY				

No Target	CR	No	CR	Normalisation of specified tumour markers AND lymph nodes < 10mm
No Target	Non-CR/non-PD	No	Non-CR/non-PD	
No Target	Not all evaluated	No	NE	
No Target	Unequivocal PD	Any	PD	
No Target	Any	Yes	PD	

<u>Note</u>: Participants with a global deterioration of health status requiring discontinuation of treatment without objective evidence of disease progression at that time should be reported as "*symptomatic deterioration*". This is a reason for stopping therapy, but is NOT objective PD. Every effort should be made to document the objective progression even after discontinuation of treatment.

*Confirmation of a complete or partial response will be undertaken based on the results of the next scan performed (i.e. no additional scans to those presented in the Schedule of Assessments in Section 6.1 are required).

4 <u>Response Duration</u>

Response duration will be measured from the time measurement criteria for CR/PR (whichever is first recorded) are first met until the first date that recurrent or progressive disease is objectively documented, taking as reference the smallest measurements recorded on study (including baseline).

5 <u>Stable Disease Duration</u>

Stable disease duration will be measured from the time of start of treatment (or randomisation for randomised studies) until the criteria for progression are met, taking as reference the smallest sum on study (including baseline).

6 <u>Methods of Measurement</u>

The same method of assessment and the same technique should be used to characterise each identified and reported lesion at baseline and during follow-up. Assessments should be identified on a calendar schedule and should not be affected by delays in therapy, which may be treatment arm dependent, unless the protocol specifies otherwise. While on study, all lesions recorded at baseline should have their actual measurements recorded at each subsequent evaluation, even when very small (e.g. 2 mm). If it is the opinion of the radiologist that the lesion has likely disappeared, the measurement should be recorded as 0 mm. If the lesion is believed to be present and is faintly seen but too small to measure, a default value of 5 mm should be assigned. For lesions which fragment/split add together the longest diameters of the fragmented portions; for lesions which coalesce, measure the maximal longest diameter for the "merged lesion".

6.1 <u>*Clinical Lesions*</u>. Clinical lesions will only be considered measurable when they are superficial and ≥ 10mm as assessed using callipers (e.g. skin nodules). For the case of skin lesions, documentation by colour photography including a ruler to estimate the size of the lesion is recommended. If feasible, imaging is preferred.

6.2 <u>*Chest X-ray*</u>. Chest CT is preferred over chest X-ray, particularly when progression is an important endpoint, since CT is more sensitive than X-ray, particularly in identifying new lesions. However, lesions ≥ 20 mm on chest X-ray may be considered measurable if they are clearly defined and surrounded by aerated lung.

6.3 *CT, MRI*. CT is the best currently available and reproducible method to measure lesions selected for response assessment. This guideline has defined measurability of lesions on CT scan based on the assumption that CT slice thickness is 5 mm or less. When CT scans have slice thickness greater than 5 mm, the minimum size for a measurable lesion should be twice the slice thickness. MRI is also acceptable in certain situations (e.g. for body scans). While PET scans are not considered adequate to measure lesions, PET-CT scans may be used providing that the measures are obtained from the CT scan and the CT scan is of identical diagnostic quality to a diagnostic CT (with IV and oral contrast).

6.4 *Ultrasound*. Ultrasound is not useful in assessment of lesion size and should not be used as a method of measurement. If new lesions are identified by ultrasound in the course of the study, confirmation by CT is advised.

6.5 *Endoscopy, Laparoscopy*. The utilisation of these techniques for objective tumour evaluation is not advised. However, they can be useful to confirm complete pathological response when biopsies are obtained or to determine relapse in trials where recurrence following complete response or surgical resection is an endpoint.

6.6 *Tumour Markers*. Tumour markers alone cannot be used to assess objective tumour response. If markers are initially above the upper normal limit, however, they must normalise for a participant to be considered in complete response.

6.7 *Cytology, Histology*. These techniques can be used to differentiate between PR and CR in rare cases if required by protocol (for example, residual lesions in tumour types such as germ cell tumours, where known residual benign tumours can remain). When effusions are known to be a potential adverse effect of treatment (e.g. with certain taxane compounds or angiogenesis inhibitors), the cytological confirmation of the neoplastic origin of any effusion that appears or worsens during treatment when the measurable tumour has met criteria for response or stable disease is advised to differentiate between response or stable disease and progressive disease.

©NHMRC Clinical Trials Centre
