# Supplementary material for: Imatinib alternating with regorafenib compared to imatinib alone for the first-line treatment of advanced gastrointestinal stromal tumor: The AGITG ALT-GIST intergroup randomized phase II trial
Source: Br J Cancer. 2025 Mar 25;132(10):897–904. doi: 10.1038/s41416-025-02983-w (PMC12081743; doi:10.1038/s41416-025-02983-w)
Supplement: Supplementary file 7 — ALT GIST HREC notification. [file 41416_2025_2983_MOESM7_ESM.pdf]

NHMRC Clinical Trials Centre
University of Sydney

ALT GIST

A randomised phase II trial of imatinib alternating with regorafenib compared to imatinib alone for the first line treatment of advanced gastrointestinal stromal tumour (GIST)

30 Mar 2017

Ethics Secretariat
Sydney Local Health District Human Research Ethics Committee
c/- Research Development Office
Royal Prince Alfred Hospital
Missenden Road, Camperdown NSW 2050

Dear Lesley:

RE: Protocol No X14-0218 & HREC/14/RPAH/286 ALT GIST - A randomised phase II trial of imatinib alternating with regorafenib compared to imatinib alone for the first line treatment of advanced gastrointestinal stromal tumour (GIST).

The following documents were submitted to the Ethics Review Committee on the 16 Jan 2017 and were subsequently approved on the 16 Feb 2017

- ALT GIST Protocol v2.0 16 Jan 2017 (Clean and tracked)
- ALT GIST PICF v3.0 16 Jan 2017 (Clean and tracked)
- ALT GIST Addendum to PICF v3.0 16 Jan 2017
- Regorafenib IB v12.0 dated 18 NOV 2016
- Imatinib Product information – dated 23rd March 2016

Thank you for taking the time to review this ALTGIST protocol amendment. The International Trial Management Group (ITMG) have discussed the ongoing recruitment difficulties and the associated funding implications and decided that they would not implement the 2:1 randomisation allocation proposed in the amendment. Therefore the ALT GIST Protocol v2.0 16 Jan 2017 and associated documents approved on the 16th Feb 2017 will not be sent to site for implementation at this stage.

For the meantime, ALT GIST sites will continue to use the previously approved ALT GIST protocol v1.0 3rd July 2014 until a subsequent protocol amendment has been developed and approved for use.

As part of the abovementioned amendment there were changes made to the PICF as a result of the Regorafenib IB update. To reduce the confusion for sites the ALT GIST PICF, ALT GIST Addendum to PICF, Regorafenib IB and Imatinib Product information will be re-submitted for review and approval in due course.

ALT GIST

A randomised phase II trial of imatinib alternating with regorafenib compared to imatinib alone for the first line treatment of advanced gastrointestinal stromal tumour (GIST)

For traceability, any future amendments to the protocol or PICF will have a subsequent version number to the above. Sites will not be sent the documents listed above, however upon acknowledgement from RPAH HREC of this letter will be sent all correspondence for their information.

Please do not hesitate to contact us if you require any further information or documentation.

Best wishes

Jenna Mitchell

Digitally signed by Jenna Mitchell
DN: cn=Jenna Mitchell, o=NHMRC CTC, ou, email=jenna.mitchell@ctc.usyd.edu.su, c=AU
Date: 2017.03.30 10:52:18 +11'00'

Jenna Mitchell | Trial Coordinator
PHONE: +61 2 9562 5329 EMAIL: jenna.mitchell@ctc.usyd.edu.au

NHMRC Clinical Trials Centre, The University of Sydney
OFFICE: The Lifehouse Building, Level 6,
119 -143 Missenden Road, Camperdown, NSW, 2050
MAIL: Locked Bag 77, Camperdown NSW 1450, Australia
FAX: +61 2 9562 5094 WEB: www.ctc.usyd.edu.au
CONNECT WITH US: TrialsCentre
